# The Nephroprotective Effect of Cornelian Cherry (*Cornus mas* L.) and Rowanberry (*Sorbus aucuparia* L.) in Gentamicin-Induced Nephrotoxicity on Wistar Rats with Emphasis on the Evaluation of Novel Renal Biomarkers and the Antioxidant Capacity in Correlation with Nitro-Oxidative Stress

**DOI:** 10.3390/nu15204392

**Published:** 2023-10-16

**Authors:** Mara Aurori, Sanda Andrei, Alexandra Iulia Dreanca, Andreea Georgiana Morohoschi, Mihaela Cotul, Mihaela Niculae, Monica Irina Nan, Andrei Răzvan Codea, Adrian Florin Gal

**Affiliations:** 1 Department of Preclinical Sciences, Faculty of Veterinary Medicine, University of Agricultural Sciences and Veterinary Medicine, 400374 Cluj-Napoca, Romania; mara.aurori@usamvcluj.ro (M.A.); alexandra.dreanca@usamvcluj.ro (A.I.D.); andreea-georgiana.morohoschi@student.usamvcluj.ro (A.G.M.); mihaela.cotul@usamvcluj.ro (M.C.); monica.nan@usamvcluj.ro (M.I.N.); adrian.gal@usamvcluj.ro (A.F.G.); 2 Department of Clinical Sciences, Faculty of Veterinary Medicine, University of Agricultural Sciences and Veterinary Medicine, 400374 Cluj-Napoca, Romania; mihaela.niculae@usamvcluj.ro (M.N.); razvan.codea@usamvcluj.ro (A.R.C.)

**Keywords:** *Cornus mas* L. fruits, *Sorbus aucuparia* L. fruits, gentamicin-induced nephrotoxicity, antioxidant capacity, oxidative stress, nitrosative stress, Cystatin C, KIM-1, iNAG

## Abstract

In spite of its well-known nephrotoxicity, gentamicin is nonetheless routinely used in humans and animals. However, no adjuvant treatments have been implemented to mitigate this harmful effect. Given this concern, medicinal plants represent a significant reservoir of natural antioxidants that could potentially reduce the renal oxidative stress induced by gentamicin. Therefore, the main objective of this research was to investigate the nephroprotective properties of *Cornus mas* and *Sorbus aucuparia* fruits in an experimental model of nephrotoxicity. The 3-week study was performed on male Wistar rats, which were randomly divided into six experimental groups, being subcutaneously treated with 50 mg/kg gentamicin and orally given *Cornus mas* and *Sorbus aucuparia* extracts, in doses of 40 mg/kg and 10 mg/kg, respectively. Antioxidant therapy significantly improved the nitro-oxidative stress parameters as well as the specific renal biomarkers KIM-1 and iNAG, demonstrating a considerable renal tubular protective impact. These outcomes were reinforced by biochemical and histopathological enhancements. Nevertheless, neither of the tested extracts succeeded in substantially diminishing BUN levels. Additionally, CysC did not significantly decline following extracts treatment, suggesting that the remedies did not effectively protect renal glomeruli against gentamicin stress. Future studies are required in order to determine the underlying mechanisms of these berries.

## 1. Introduction

Acute kidney injury (AKI), also referred to as acute renal failure, is defined as a drastic decrease in glomerular filtration rate and a rapid increase in serum creatinine level, inevitably resulting in acute uremia and alterations of urine output [1]. AKI represents a major healthcare issue around the world, involving roughly 13.3 million individuals globally each year [2]. Similarly to humans, companion animals develop AKI as well. It was discovered that cats have higher AKI-related mortality rates when compared to dogs (53.1% vs. 45%) [3]. AKI is linked to elaborate pathophysiological processes [4] and is also brought on by a wide range of intricate causes, which are categorized into prerenal, renal, and postrenal AKI [2]. However, despite the broad spectrum of AKI occurrences, one of the main causes of this condition, which affects both humans and animals, is represented by nephrotoxicity.

As such, nephrotoxicity can be described as any form of renal damage caused by overdoses of medicines and toxins. Worryingly, 15% of AKI cases diagnosed in ICUs (Intensive Care Units) are caused by drug-induced nephrotoxicity, being the third most common cause of AKI [5]. Drugs may induce glomerular injury, interstitial nephritis, and/or acute tubular damage as a result of direct nephrotoxicity, as well as a decrease in renal perfusion as a result of indirect nephrotoxicity [2]. The most frequent type of drug-induced nephrotoxicity seen in ICUs is acute tubular necrosis, which is largely linked to certain antibiotics, chemotherapeutics, antifungals, antivirals, contrast media, and other drug classes. [6]. In a nearly identical manner, for canines and felines, nephrotoxic substances are likely the main source of AKI’s unfavorable consequences. The most dangerous primary cause of acute kidney injury is ethylene glycol poisoning, which has a mortality rate of almost 100%. Compared to dogs, cats are substantially more sensitive to drug-induced nephrotoxicity in terms of prevalence [3,7]. Nonetheless, one of the most well-known groups of antibiotics that generate nephrotoxicity, in both humans and animals, is represented by aminoglycosides, with researchers showing a particular interest in gentamicin’s nephrotoxicity.

Gentamicin is an antimicrobial drug that is used in clinical settings due to its broad range of activity against Gram-negative pathogens, such as *Proteus*, *Pseudomonas*, *Klebsiella*, *Escherichia*, and *Serratia*. The glomerular filtration process entirely removes unaltered gentamicin from the organism, predisposing it to accumulate in the renal cortex. As a result of the accumulation at the renal level and the establishment of various complex pathological processes, nephrotoxicity is a well-known adverse effect of this antibiotic, which is regarded as the most nephrotoxic antibiotic of the aminoglycoside class, displaying severe impacts even at low doses. As such, gentamicin-induced nephrotoxicity is characterized by its absorption in the renal proximal convoluted tubule cells, specifically in the lysosomes, where it determines changes in the enzymatic processes, which may further induce a simple loss of cells’ brush border or, in more severe instances, an acute tubular necrosis [8,9,10,11]. Furthermore, it is believed that oxidative stress plays a crucial part in the nephrotoxicity caused by gentamicin. Thus, the formation of reactive oxygen species (ROS) by renal cortical mitochondria, namely hydrogen peroxide (H_2_O_2_), hydroxyl radical (OH•), and superoxide anion (O_2_^−•^), along with a rise in lipid peroxidation and protein oxidation, are all linked to gentamicin and ultimately result in renal structural and functional decline. Moreover, it has been shown experimentally that the production of ROS causes a decrease in the endogenous antioxidant defense mechanisms, including a reduction in the activity of the antioxidant enzymes superoxide dismutase (SOD), catalase (CAT), and glutathione peroxidase (GPx) [9,10,12]. In light of this worry, natural reserves such as medicinal herbs represent a valuable source of natural antioxidants that can be utilized as therapeutics to alleviate the renal toxicity induced by pharmaceuticals that promote oxidative stress [10].

One such medicinal herb might be *Cornus mas* L., also referred to as European cornelian cherry or dogwood, that is part of the *Cornaceae* family. It is a broadleaf tree that is widely spread over the Caucasus and Southeast Europe [13,14]. In traditional medicine, *Cornus mas* L. fruits have been utilized for centuries in the treatment of various ailments [15,16]. In accordance with previously published research, a wealth of biologically active chemical compounds has been identified in these berries. As such, anthocyanins, iridoids, phenolic acids, flavonoids, and tannins are the most commonly described organic compounds present in *Cornus mas* L. fruits [17]. Therefore, these fruits have been the focus of several studies, demonstrating that they possess multiple biological activities [18], the most important of which is the antioxidant effect. This property was evaluated in both in vitro [19,20,21] and in vivo models [22,23,24], indicating a protective impact against the harmful effects of oxidative stress encountered in various pathologies.

*Sorbus aucuparia* L., commonly known as European rowanberry or mountain ash, is a deciduous shrub widely grown in the European continent, belonging to the *Rosaceae* family. Due to their well-known high nutritional content, these fruits have been used in folk medicine to cure a variety of diseases [25,26,27]. According to earlier studies regarding the phytochemical profiling, it was revealed that *Sorbus aucuparia* L. fruits possess an increased quantity of organic acids (particularly ascorbic acid), polyphenols (especially phenolic acids—chlorogenic and neochlorogenic acids), carotenoids, and microelements. However, its phytochemical characterization remains incomplete, as future studies are required [25,28]. Furthermore, the biological activities of *Sorbus aucuparia* L. have received very little research, although antioxidant activity has been identified as a key component of their health-promoting abilities. Nevertheless, this property has been investigated in a limited number of in vitro studies, such as scavenging experiments using artificial free radicals [25,27,29]. Further research into its biological processes is therefore required, perhaps even including in vivo experiments, with an emphasis on its antioxidant qualities in relation to oxidative stress.

Following earlier research [30] that indicated a significant cytoprotective impact of *Cornus mas* L. fruits in gentamicin-induced nephrotoxicity on primary renal epithelial cells in vitro, we chose to further the research by examining the potential nephroprotective effect of *Cornus mas* L. fruits in the context of gentamicin-induced nephrotoxicity in a rat experimental model. Furthermore, given that the fruits of *Sorbus aucuparia* L. were discovered to contain a variety of important active constituents and that recent in vitro studies suggested they have a significant antioxidant capacity, we felt it was essential to extensively evaluate the antioxidant and nephroprotective benefits of these fruits, as, to the best of our knowledge, there have been no previous in vivo studies discussing their great antioxidant potential in kidney disease. Thus, the aim of this paper was to investigate the nephroprotective effect of *Cornus mas* L. and *Sorbus aucuparia* L. fruits in gentamicin-induced nephrotoxicity in a rat experimental model, with a focus on their antioxidant properties in connection with oxidative stress, which may contribute to the creation of novel medications to prevent the onset or slow the evolution of drug-induced renal injury.

## 2. Materials and Methods

### 2.1. Reagents and Chemicals

Elabscience Biotechnology Inc., Texas, HT, USA and AMEDA Labordiagnostik GmbH, Graz, Austria provided the analysis kits that were employed in this experiment. Gentamicin injectable solution was supplied by Dopharma, Timişoara, Romania. The additional chemicals were purchased from Merck and Sigma Aldrich, both of Darmstadt, Germany.

### 2.2. Collection of Plant Components and Arrangement of Fruit Samples

On the hillsides of Mărişel commune, Cluj County, Romania (46°40′03.7″ N 23°06′35.6″ E), *Cornus mas* L. and *Sorbus aucuparia* L. trees were harvested for their twigs containing leaves and fruits, in August–September 2021. After plant identification, the twigs, leaves, and seeds were manually detached and the total quantity of fruit kernels was divided into sample packets, each holding approximately 100 g, and refrigerated at −18 °C for further analysis.

### 2.3. Preparation of Cornus mas L. and Sorbus aucuparia L. Fruit Extracts

Firstly, both types of fruits were defrosted and dried using a TEESA TSA3031 dehumidifier (Lechpol Electronics Leszek Sp. K., Garwolin, Poland) set at 45 °C for seven days and further ground into a fine powder. In the case of *Cornus mas* L. fruits, an ethanolic extract was prepared. As such, 30 g of fruit powder together with 300 mL of 96% ethanol were employed for the extraction. A magnetic centrifuge operating at 1000 rpm was used to homogenize the resultant mixture for two hours. Following centrifugation, the mixture was filtered, and the powder residuum was extracted repeatedly by employing the same techniques. Additionally, using an Eppendorf Concentrator Plus evaporator (Eppendorf, Hamburg, Germany) set at 45 °C, the resultant extracts were combined and concentrated by evaporation, up until the entire volume of ethanol was vaporized. Lastly, the obtained concentrated fruit extract was diluted in distilled water and further used for direct measurements and experimental protocols.

In order to establish the dosage of the *Cornus mas* L. fruit extract to be administered to the rodents, the total polyphenolic content was considered, which was measured using the Folin–Ciocâlteu method. As a result, an indicated dose of 40 mg polyphenols/kg b.w. (corresponding approximately to 13.5 mg polyphenols/animal) was employed for the experiment, as a similar amount had previously been determined to be harmless [31]. Moreover, the extract dose needed for each individual was calculated in mL based on their weight. Finally, after determining the overall quantity required, the extraction was carried out repeatedly until the necessary amount of phenolic compounds was obtained.

Regarding *Sorbus aucuparia* L. fruits, because information from the reviewed literature suggested that these berries are rich in lipophilic compounds [27,32], particularly carotenoids, which have a much lower solubility in alcohol, we believed it would be intriguing to perform a specific carotenoid extraction. As a result, 75 g of fruit powder was extracted using a 270 mL mixing solution of methanol: ethyl acetate: petroleum ether 1:1:1 (*v*/*v*/*v*). Following the 2 h homogenization, straining, and powder sediment re-extraction, the appropriate filtrates were pooled in a separator funnel and further sequentially partitioned with water, diethyl ether, and saturated NaCl solution. Moreover, the organic phase was isolated and put through anhydrous Na_2_SO_4_ to remove the remaining water and was later exposed to rotational evaporation at 40 °C. The remaining oleoresin was dissolved in Medium-Chain Triglycerides (MCT) oil. This excipient has previously been used and proven to be safe [33]. The final extract was employed for subsequent investigations and experimental protocols.

Similar to *Cornus mas* L., the dose of *Sorbus aucuparia* L. fruit extract was established by measuring total carotenoids, through a spectrophotometric assay, and by reviewing previously published studies [34,35]. As such, a suggested dose of 10 mg total carotenoids/kg b.w. (being almost equivalent to 8.75 mg total carotenoids/animal) was utilized for the in vivo study. For oral administration, the extract dose was adjusted in milliliters for each individual. The extraction was performed until the entire quantity of total carotenoids needed was obtained.

### 2.4. Experimental Rodents

The study was carried out at the Cluj-Napoca, Romania, Faculty of Veterinary Medicine’s accredited Animal Research Center. Forty-eight adult male Wistar rats employed for the experiment, weighing approximately 300 g, were purchased from the Institutional Biobase of the University of Medicine and Pharmacy “Iuliu Haţieganu”, Cluj-Napoca, Romania. The housing conditions were arranged in accordance with Ordinance 63/2010/EU and ISO 10993-6 specifications [36]. As such, after randomly dividing the animals into 6 equal groups of 8 individuals each, they were placed and maintained in air-conditioned cages with a 12 h diurnal cycle, at an ambient temperature of 25 ± 2 °C and a humidity level of 55 ± 10%. Also, the individuals had access to conventional rodent food acquired from the Cantacuzino Institute of Bucharest, Romania, and fresh tap water ad libitum. The subjects were acclimatized in this setting for one week prior to the experimental protocols. Importantly, all animal procedures complied with the requirements of State Law No. 43/2014 and Ordinance 63/2010. The research was approved by the Ethics Committee of the University of Agricultural Sciences and Veterinary Medicine, Cluj-Napoca, Romania (no. 256/21.04.2021) as well as the Regional Sanitary Veterinary and Food Safety Authority (no. 274/12.11.2021). Also, the application of surgical methods complied with ISO 10993-6 [36].

### 2.5. Experimental Design

A number of 48 mature male Wistar rats were randomly assigned to one of the six following groups:

Group I—Control (*n* = 8): received 0.5 mL saline solution (NaCl) 0.9% subcutaneously for 21 days;

Group II—Gentamicin (*n* = 8): received 50 mg/kg gentamicin (GentaJect 10% ^®^, Dopharma, Romania) subcutaneously for 11 days followed by 0.5 mL NaCl 0.9% subcutaneously for another 10 days;

Group III—*Cornus mas* L. (*n* = 8): received 40 mg/kg *Cornus mas* L. fruit extract orally for 21 days;

Group IV—*Sorbus aucuparia* L. (*n* = 8): received 10 mg/kg *Sorbus aucuparia* L. fruit extract orally for 21 days;

Group V—Gentamicin + *Cornus mas* L. (*n* = 8): received 50 mg/kg gentamicin subcutaneously and 40 mg/kg *Cornus mas* L. fruit extract orally for 11 days followed by 0.5 mL NaCl 0.9% subcutaneously and 40 mg/kg *Cornus mas* L. fruit extract orally for another 10 days;

Group VI—Gentamicin + *Sorbus aucuparia* L. (*n* = 8): received 50 mg/kg gentamicin subcutaneously and 10 mg/kg *Sorbus aucuparia* L. fruit extract orally for 11 days followed by 0.5 mL NaCl 0.9% subcutaneously and 10 mg/kg *Sorbus aucuparia* L. fruit extract orally for another 10 days.

During the study, survival rate, pain, infection establishment, urine production and water intake were all thoroughly monitored. On days 1, 11, and 21, measurements of body mass along with collection of blood and urine samples were performed. Consequently, under general anesthesia, complete blood samples were obtained from the infraorbital sinus and placed in EDTA (Ethylene Diamine Tetra-Acetic Acid) and heparin-coated micro tubes for hematological and biochemical analyses, respectively. An amount of approximately 1–2 mL of blood was collected from each individual. Centrifugation (3000 rpm for 5 min) was used to prepare the heparin tubes in order to extract the plasma needed for further dosing. Moreover, after trimming and cleaning the ventral abdominal area, an ultrasound-guided cystocentesis was used to collect urine samples. They were placed in sterile Eppendorf tubes and further centrifuged at 1000 rpm for 5 min. After centrifugation, the supernatant was employed for subsequent determinations.

On the 22nd day of the experimental period, all subjects were painlessly sacrificed through cervical dislocation while under general anesthesia, in conformity with international protocols [37]. Following this procedure, abdominal incisions were performed in order to excise kidney and liver samples for further tissue biochemical analysis. A part of each of these samples were cleaned with phosphate-buffered saline solution and fixed in paraformaldehyde for subsequent histopathological examination.

### 2.6. Blood Analysis

#### 2.6.1. Hematological Analysis

An automated Abaxis VetScan HM5 hematology analyzer (Abaxis Inc., Union City, CA, USA) was used to perform the hematological analyses on whole blood to determine the complete blood count: white blood cells (WBS), lymphocytes (LYM), monocytes (MON), neutrophils (NEU), red blood cells (RBC), hemoglobin (HGB), hematocrit (HCT), and platelet count (PLT).

#### 2.6.2. Biochemical Analysis

An automated Element RC clinical biochemistry analyzer (Scil Animal Care Company, Altorf, France) was employed for performing biochemical analyses on whole blood in order to determine the following parameters: total proteins (PT), albumin (ALB), globulin (GLOB), alanine aminotransferase (ALT), alkaline phosphatase (ALP), glucose (GLU), blood urea nitrogen (BUN), and creatinine (CREA).

#### 2.6.3. Rat Cystatin C (CysC) Activity Assay

Cystatin C activity was determined using a quantitative ELISA assay kit (Elabscience Biotechnology Inc., Houston, TX, USA). The principle of assessment involves the quantitative sandwich enzyme immunoassay technique. This measurement was assessed in line with the instructions provided in the kit. The absorbance was measured spectrophotometrically at a wavelength of 450 nm. A standard curve with six distinct values ranging from 0.09 to 2.81 ng/mL was obtained for calculation. The results were expressed as ng/mL.

#### 2.6.4. Rat Kidney Injury Molecule 1 (KIM-1) Activity Assay

Similar to Cystatin C, a quantitative ELISA assay kit (Elabscience Biotechnology Inc., Houston, TX, USA) was employed for measuring Kidney Injury Molecule 1. This assay involves the sandwich ELISA principle. The guidelines provided in the kit were followed for the assessment. At a wavelength of 450 nm, the absorbance was measured. For calculation, a standard curve with six concentration levels, in the range of 0.12–1.22 ng/mL, was obtained. The outcomes were presented in ng/mL.

#### 2.6.5. Markers of Nitro-Oxidative Stress

- Catalase (CAT) Activity Assay

CAT activity was determined using a colorimetric assay kit (Catalase (CAT) Activity Assay Kit—Elabscience Biotechnology Inc., Houston, TX, USA). The detection principle is defined as the ability of catalase to interact with hydrogen peroxide, as the remaining H_2_O_2_ combines with ammonium molybdate to generate a yellow complex, which can be detected spectrophotometrically at 405 nm. The assay protocol was conducted in accordance with the kit’s instructions. The spectrophotometric measurement was performed using the SPECTROstar^®^ Nano spectrophotometer (BMG Labtech, Ortenberg, Baden-Württemberg, Germany). The outcomes were expressed as U/mL.

- Superoxide Dismutase (SOD) Activity Assay

SOD analysis was carried out using a similar colorimetric assay kit (Total Superoxide Dismutase (T-SOD) Activity Assay Kit (Hydroxylamine Method)—Elabscience Biotechnology Inc., Houston, TX, USA). The method’s principle is based on the capacity of SOD to inhibit superoxide anion free radicals, which in turn inhibits the formation of nitrite (purple color complex), as the intensity of the color is reversely proportional to the quantity of the antioxidant enzyme. The samples were prepared following the instructions of the manufacturer. The samples’ absorbances were measured in contrast with blank (distilled water) and standard samples, at 550 nm, using the spectrophotometer mentioned above. The results were calculated based on a mathematical formula described in the kit’s instructions and were further given in U/mL.

- Glutathione Peroxidase (GPx) Activity Assay

A Glutathione Peroxidase (GSH-Px) Colorimetric Assay Kit (Elabscience Biotechnology Inc., Houston, TX, USA) was employed for measuring glutathione peroxidase. The kit’s principle is based on the detection of reduced glutathione (GSH), involved in both enzymatic (catalyzed by GSH-Px) and non-enzymatic reactions, and its spectrophotometric measurement at 412 nm. The assay’s operation steps, measurements and calculations were conducted according to the manufacturer’s instructions. GPx activity was expressed as U.

- Total Antioxidant Capacity (TAC) Activity Assay

TAC was assessed employing a commercially available colorimetric kit purchased from Elabscience Biotechnology Inc., Houston, TX, USA. The fundament of this assay is represented by the capability of antioxidants to reduce Fe^3+^ to Fe^2+^, which further forms, together with phenanthroline substance, stable complexes. The instructions provided in the kit were followed carefully for assessment. Each sample’s absorbance was measured at 520 nm against blank (distilled water) and control. The measurements were performed using the SPECTROstar^®^ Nano—BMG Labtech, Ortenberg, Baden-Württemberg, Germany. The data were provided in U/mL.

- Malondialdehyde (MDA) Activity Assay

MDA was measured using an assay kit from Elabscience Biotechnology Inc., Houston, Texas, USA (Malondialdehyde, MDA, Colorimetric Assay Kit). The approach is based on the interaction of MDA with thiobarbituric acid (TBA), which results in the creation of an MDA-TBA complex that can be quantified spectrophotometrically. The preparation of the samples followed the kit’s instructions. Using the earlier mentioned spectrophotometer, the absorbance of the samples was evaluated in comparison to a blank (distilled water) at 532 and 600 nm, respectively. The difference between the absorbance at 532 nm and at 600 nm was used to compute the final absorbance. The results were given in nmoL/mL.

- Nitric Oxide (NO) Activity Assay

The NO Colorimetric Assay Kit (Elabscience Biotechnology Inc., Houston, TX, USA) is a commercial kit used to measure NO levels. The principle of assessment involves the oxidation of NO in aqueous solution and the subsequent formation of NO_2_^−^, which combines with the chromogenic agent and further forms a crimson compound. By determining the compound’s absorbance at 550 nm, the concentration of NO can be indirectly determined. Samples were prepared in accordance with the producer’s instructions. Samples’ absorbances were measured using 1 cm cuvettes and the SPECTROstar^®^ Nano spectrophotometer. The data were ultimately expressed in µmol/L.

### 2.7. Urine Analysis

#### Urinary N-Acetyl-*β*-D-Glucosaminidase Index (iNAG) Activity

*N*-Acetyl-*β*-D-Glucosaminidase (NAG) activity was determined using a commercially available colorimetric assay kit obtained from Elabscience Biotechnology Inc., Houston, TX, USA. The methodology principle is based on the ability of the NAG enzyme to hydrolyze 4-Nitrophenyl *N*-Acetyl-*β*-D-Glucosaminide substrate, with the release of *p*-nitrophenol, which can be measured spectrophotometrically at 400 nm. Following the kit’s instructions regarding the assay protocol, measurements, and calculation, urinary NAG concentrations were expressed as U/L.

Urinary creatinine was assessed using a colorimetric assay kit (AMEDA Labordiagnostik GmbH, Wien, Austria), based on the Jaffe reaction. The samples were prepared according to the instructions in the kit. The samples’ absorbances were measured using the spectrophotometer mentioned previously, at a wavelength of 510 nm. The results were further provided in g/L.

The NAG index was further calculated utilizing the results obtained from urinary NAG and creatinine assessments, employing the following equation [38]:

iNAG (U/g) = urinary NAG (U/L)/urinary creatinine (g/L)

### 2.8. Tissue Analysis

#### 2.8.1. Preparation of Kidney and Liver Tissue Homogenates

The protocol for obtaining the tissue extracts was performed in accordance with Evelson et al. [39], with slight modifications. Following washing for excess blood removal and weighing, the tissue samples were homogenized in PBS (0.01 mM, pH 7.34) at a tissue–buffer ratio of 1 g/9 mL. The homogenates were then centrifuged at 10 × 10,000 rpm for 2 min and the supernatant was used for further determinations.

#### 2.8.2. Total Proteins

After obtaining the kidney and liver tissue homogenates, total protein concentration was assessed using the Biuret method [40]. The absorbances were measured at 555 nm using the SPECTROstar^®^ Nano (BMG Labtech) spectrophotometer. The results were expressed as mg protein/g tissue.

#### 2.8.3. Markers of Nitro-Oxidative Stress

Catalase (CAT), superoxide dismutase (SOD), glutathione peroxidase (GPx), total antioxidant capacity (TAC), malondialdehyde (MDA), and nitric oxide (NO) were analyzed from tissue homogenates, using the same analysis kits purchased from Elabscience Biotechnology Inc., Houston, TX, USA, as previously described in plasma analyses. The outcomes were presented in U/g tissue, nmoL/g tissue, and µmol/g tissue, respectively.

### 2.9. Histopathological Examination

Fragments of kidney and liver tissue were sectioned into 4 mm thick slices and preserved in 10% formalin for five days. They were subsequently treated with three 1-butanol baths followed by progressive dehydration with 70%, 96%, and 100% ethyl alcohol for one hour each. Following fixation, samples were prepared for histology by being embedded in paraffin, cut into 5 µm sections using a Leica rotary microtome (RM2125, Wetzlar, Germany), and stained with Goldner’s trichrome. An Olympus BX-41 microscope coupled with an Olympus E330 camera (Olympus, Tokyo, Japan) was used to evaluate the slides. The observed modifications were scored as follows:

- Renal tissue:

0 = unmodified histological aspect

1 = zonal septal congestion with isolated vacuolar degenerative lesions

2 = zonal septal congestion, vacuolar degeneration, and isolated apoptosis of the tubular epithelium

3 = diffuse septal congestion, glomerular congestion, multifocal vacuolation, and apoptosis of the tubular epithelium

4 = coagulative necrosis of the tubular epithelium along with the presence of proteinaceous material in the lumen of the renal tubules

- Liver tissue:

0 = unaltered liver parenchyma

1 = localized vacuolar degenerative lesions

2 = multifocal vacuolar degeneration with isolated hepatocyte apoptosis

3 = diffuse hepatocyte vacuolation throughout the liver lobule and isolated hepatocyte apoptosis

### 2.10. Statistical Analysis

After each assessment was conducted in three replicates, the data were presented as mean ± SD. The statistical assessment was carried out using the statistical program Graph Pad Prism 8 (San Diego, CA, USA). Data analysis was performed employing one-way ANOVA and two-way ANOVA. The threshold for significance was set at *p* < 0.05.

## 3. Results

### 3.1. General Clinical Examination and Body Weight Monitoring

**Figure 1:**
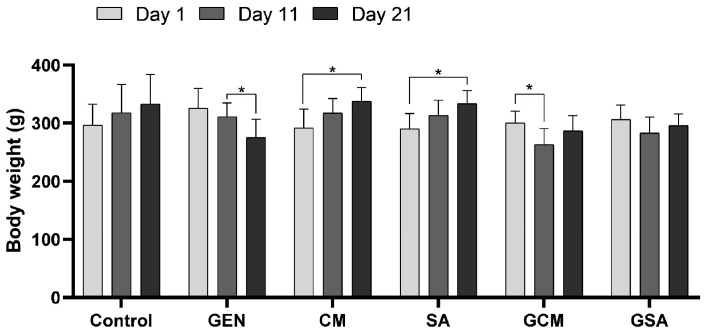
Subjects’ weight distribution throughout the study (GEN = gentamicin group; CM = *Cornus mas* L. group; SA = *Sorbus aucuparia* L. group; GCM = gentamicin + *Cornus mas* L. group; GSA = gentamicin + *Sorbus aucuparia* L. group). Statistics performed utilizing one-way ANOVA; * *p* < 0.05. The outcomes represent mean ± SD of three replicate assessments.

Throughout the experiment, the subjects’ overall health was carefully monitored and some changes were recorded. Consequently, piloerection and anorexia were noticed in the GEN group, while none of these symptoms were noted in the other groups, proving that the herbal medication had neither harmful nor deleterious effects. However, no rats showed signs of pain, infection establishment, altered urine output, reduced water intake, or decreased appetite. Additionally, three weighings of the individuals were performed, as shown in Figure 1.

The Control group did not show any significant fluctuations in body mass. The gentamicin-treated rats recorded a body weight of 325.79 ± 34.26 g on day 1, 311 ± 23.94 g on day 11, and 275.06 ± 31.64 g on day 21. Thus, a significant decrease in body mass was observed from day 11 to day 21 (*p* < 0.05). Comparatively to the other groups, this group recorded the lowest body weight before euthanasia. Furthermore, in the *Cornus mas* L. and *Sorbus aucuparia* L. extract-treated groups, a significant increase in body weight was observed from day 1 to the last day (*p* < 0.05), with rats increasing from 291.75 ± 32.38 g to 337.5 ± 24.10 g and from 290.08 ± 26.36 g to 333.83 ± 22.33 g, respectively. Further, while no significant weight fluctuations were observed in the gentamicin and *Sorbus aucuparia* L. extract-treated subjects, the gentamicin and *Cornus mas* L. extract-treated group showed a significant decrease in body weight from day 1 (300.36 ± 20.39 g) until day 11 (262.79 ± 27.90 g) (*p* < 0.05). Subsequently, individuals showed an increase in weight on day 21 (286.69 ± 26.36 g), without statistical significance. This could be attributed to the protective effect of *Cornus mas* L. extract that gradually set in following the acute kidney injury caused by gentamicin.

### 3.2. Hematological Profile

**Figure 2:**
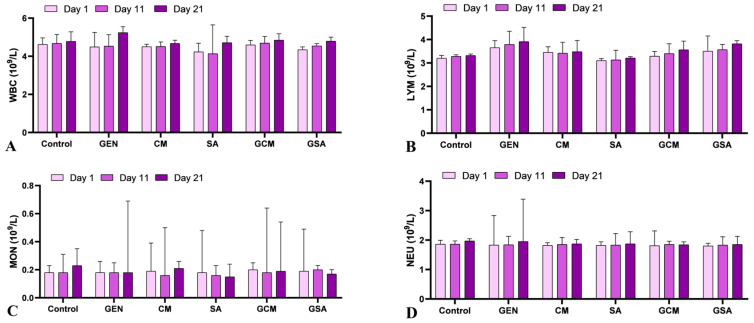

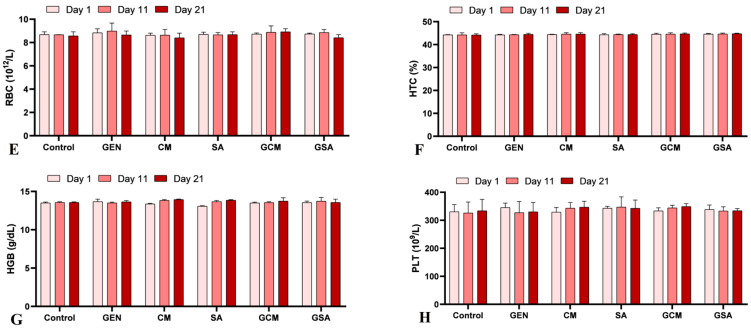
Hematological Profile. (**A**) White blood cells. (**B**) Lymphocytes. (**C**) Monocytes. (**D**) Neutrophils. (**E**) Red blood cells. (**F**) Hematocrit. (**G**) Hemoglobin. (**H**) Platelets. Statistics performed utilizing one-way ANOVA and two-way ANOVA; *p* > 0.05. The findings indicate mean ± SD of triplicate measurements.

The complete blood count reflected normal values in all groups, being similar to those found in the Control group, with no statistically significant variations. Figure 2 displays the hematological profile’s outcomes.

### 3.3. Biochemical Profile

**Figure 3:**
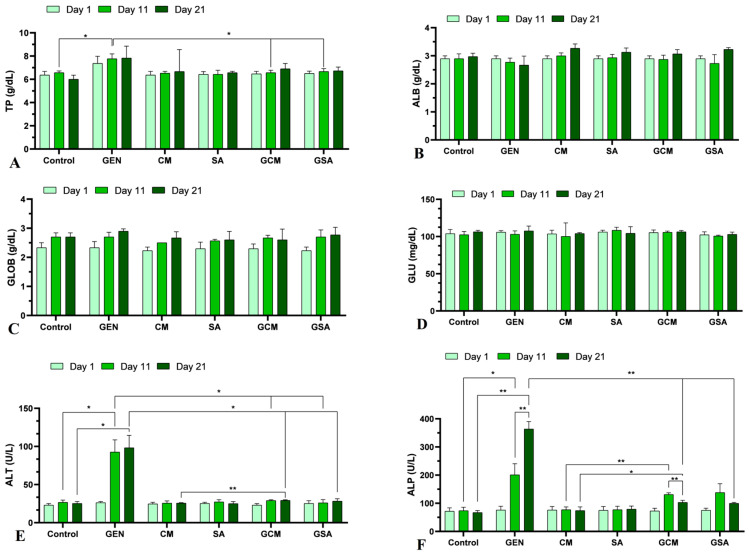

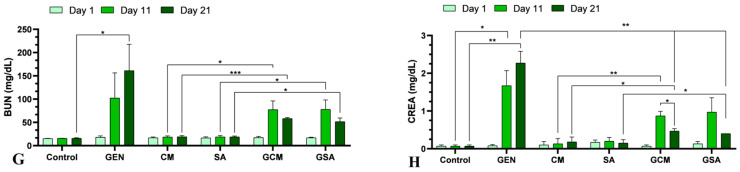
Biochemical profile. (**A**) Total Proteins. (**B**) Albumin. (**C**) Globulins. (**D**) Glucose. (**E**) Alanine aminotransferase. (**F**) Alkaline phosphatase. (**G**) Blood urea nitrogen. (**H**) Creatinine. Statistics performed utilizing one-way ANOVA and two-way ANOVA; * *p* < 0.05, ** *p* < 0.01, *** *p* < 0.001. The results are expressed as mean ± SD of three replicate measurements.

Regarding the biochemical profiling, analyses were performed on whole blood using biochemical rotors in order to determine the parameters that are further presented in detail in Figure 3.

As such, total proteins were significantly increased in the GEN group compared to the Control group on the 11th day of the experiment (7.77 ± 0.42 vs. 6.57 ± 0.15 g/dL, *p* < 0.05). Improvements in total proteins were observed in rats treated with extracts and the antibiotic, as they were significantly reduced compared to the individuals treated with the antibiotic alone (*p* < 0.05). Moreover, the PT levels showed similarity to those of the extract-treated groups (*p* > 0.05). As for albumin, globulin, and glucose levels, no significant changes were recorded, being within the reference ranges [41]. These outcomes can be followed graphically in Figure 3A–D.

Alanine aminotransferase (ALT) levels were significantly increased in the GEN group in comparison to the Control group on both day 11 (92.67 ± 16.07 vs. 26.67 ± 3.06 U/L) and day 21 (98.33 ± 16.17 vs. 25.33 ± 2.52 U/L) (*p* < 0.05). Importantly, ALT registered a significant decrease in the extract and antibiotic-treated groups in comparison to the antibiotic-treated rats (*p* < 0.05), reaching values of 29 ± 1.0 U/L on day 11 and 29.25 ± 0.86 U/L on day 21 in the GCM group, and values of 26 ± 4.36 U/L on day 11 and 28.33 ± 3.06 U/L on day 21 in the GSA group. However, on day 21, the subjects treated with gentamicin and *Cornus mas* L. extract registered significant elevations in ALT levels when compared to the rats treated with extract alone (vs. 25.67 ± 0.58 U/L, *p* < 0.01). On the other hand, ALT levels in the group receiving gentamicin and *Sorbus aucuparia* L. extract were not significantly distinct from those in the group receiving the extract alone (vs. 252.65 U/L, *p* > 0.05). Figure 3E illustrates these results graphically.

Alkaline phosphatase (ALP) showed a significant increase in the gentamicin group compared to the Control group starting on day 11 (*p* < 0.05), and continuing until the end of the experiment (*p* < 0.01). Additionally, ALP registered a substantial rise in the GEN group from one monitoring to another (*p* < 0.01), being on the first day 76.33 ± 13.2 U/L, on day 11 201.33 ± 39.27 U/L, and reaching on day 21 the value of 363.67 ± 26.54 U/L. By comparison of this group to the GCM and GSA groups, their alkaline phosphatase significantly decreased on the last day, with the extract + antibiotic-treated rats registering values of 103.33 ± 7.09 U/L and 99.67 ± 3.51 U/L, respectively (*p* < 0.01). In the GSA group, there were no significant changes in ALP levels between the monitored days, showing a protective effect similar to that of the group treated with the extract alone (*p* > 0.05). However, a significant decrease in ALP levels was observed in the GCM group from day 11 to day 21 (131 ± 6.08 vs. 103.33 ± 7.09 U/L, *p* < 0.01), reflecting the gradual onset of the protective effect of *Cornus mas* L. extract. In contrast, when compared to the CM group, ALP values were significantly increased on both day 11 (vs. 77.33 ± 9.71 U/L, *p* < 0.01) and day 21 (vs. 74 ± 13.11 U/L, *p* < 0.05). This could be attributed to the requirement of administering the extract over an extended period of time or at a higher dose. These outcomes are displayed in Figure 3F.

Blood urea nitrogen (BUN) was determined to have risen significantly in the gentamicin-treated experimental animals compared to the saline-treated rats on the last day of the experiment (161.24 ± 56.44 vs. 15.64 ± 1.33 mg/dL, *p* < 0.05). The BUN increase in the gentamicin-exposed group could be attributed to both renal and hepatic impairment, or dehydration. Furthermore, no significant differences were registered between the groups treated with extract and antibiotic and the group treated with antibiotic alone (*p* > 0.05). Contrarily, significant increases in BUN levels were registered in the GCM group compared to the CM group on day 11 (77.4 ± 8.67 vs. 18.56 ± 2.33 mg/dL, *p* < 0.05), as well as on day 21 (58.14 ± 2.21 vs. 19.07 ± 2.64 mg/dL, *p* < 0.001). Similarly to the GCM group, the GSA group exhibited significantly higher BUN levels than the SA group both on day 11 (77.96 ± 20.19 vs. 18.95 ± 2.80 mg/dL, *p* < 0.05) and day 21 (51.61 ± 7.87 vs. 18.76 ± 2.0 mg/dL, *p* < 0.05). Figure 3G shows these results graphically.

In terms of creatinine (CREA) analysis, there was a significant increase in creatinine levels in the GEN group compared to the Control group on both day 11 (1.67 ± 0.4 vs. 0.07 ± 0.03 mg/dL, *p* < 0.05) and day 21 (2.27 ± 0.31 vs. 0.07 ± 0.03 mg/dL, *p* < 0.01). Creatinine increased significantly from day 1 to day 11 and continued to increase, without significant difference, until day 21. This increase, thus, demonstrates renal impairment due to gentamicin administration. These results can be correlated with the results obtained in the BUN analysis. Furthermore, on the last day of the experiment, there was a significant decrease in creatinine values (*p* < 0.01) in the rats treated with extract and antibiotic (0.47 ± 0.06 mg/dL and 0.4 ± 0.0 mg/dL, respectively) compared to the subjects receiving antibiotic treatment only (2.27 ± 0.31 mg/dL). Moreover, the GCM group showed a significant decrease in creatinine from day 11 to day 21 (0.87 ± 0.12 vs. 0.47 ± 0.06 mg/dL, *p* < 0.05). However, by comparing the GCM group to the CM group, there was an increase in creatinine values on both day 11 (*p* < 0.01) and day 21 (*p* < 0.05). This rise was more pronounced on day 11 compared to day 21. Also, by comparing the GSA and SA groups, no significant differences were observed on the 1st and 11th day of the experiment. In contrast, on day 21, a significant increase in creatinine levels was recorded in the GSA group compared to the SA group (0.4 ± 0.0 vs. 0.15 ± 0.09 mg/dL, *p* < 0.05). These outcomes are displayed graphically in Figure 3H.

### 3.4. Cystatin C (CysC) Activity Results

**Figure 4:**
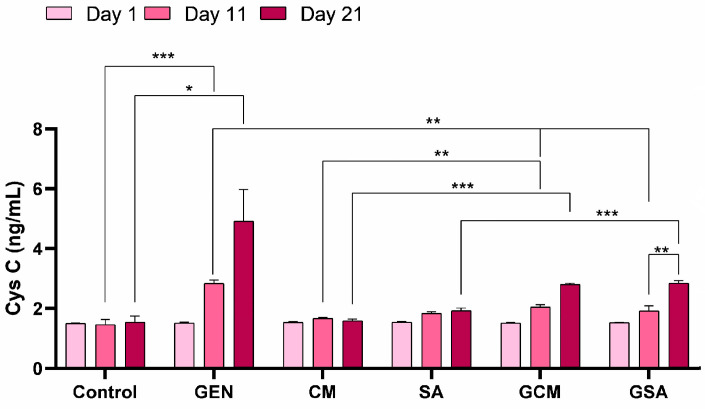
Cystatin C measurements in the experimental groups. Statistics performed utilizing one-way ANOVA and two-way ANOVA; * *p* < 0.05, ** *p* < 0.01, *** *p* < 0.001. The outcomes represent mean ± SD of three replicate analyses.

The determination of CysC activity was performed using the sandwich ELISA technique. Thus, a significant increase in CysC levels was registered in the GEN group compared to the Control group on the 11th day (2.83 ± 0.11 vs. 1.46 ± 0.16 ng/mL, *p* < 0.001), as well as on the 21st day of the experiment (4.91 ± 1.06 vs. 1.54 ± 0.21 ng/mL, *p* < 0.05). Importantly, on day 11, significant improvements were observed in the GCM (2.05 ± 0.06 ng/mL) and GSA (1.92 ± 0.16 ng/mL) groups compared to the GEN group, *p* being < 0.01. However, no significant difference in CysC levels was observed between both groups and the GEN-treated rats at the end of the trial (*p* > 0.05). Moreover, in the GSA group, a significant increase in CysC values was observed on day 21 compared to day 11 (2.84 ± 0.08 vs. 1.92 ± 0.16 ng/mL, *p* < 0.01). This group also showed a substantial increase compared to the subjects that had only received *Sorbus aucuparia* L. extract (vs. 1.92 ± 0.09 ng/mL, *p* < 0.001). Regarding the GCM group, a significant rise in CysC was observed compared to the CM group, both on day 11 (2.05 ± 0.06 ng/mL vs. 1.66 ± 0.03 ng/mL, *p* < 0.01) and on day 21 (2.80 ± 0.03 vs. 1.59 ± 0.05 ng/mL, *p* < 0.001). It was noticeable that these increases were more pronounced on day 21 than on day 11. Figure 4 shows a detailed replication of these outcomes.

### 3.5. Kidney Injury Molecule 1 (KIM-1) Activity Results

**Figure 5:**
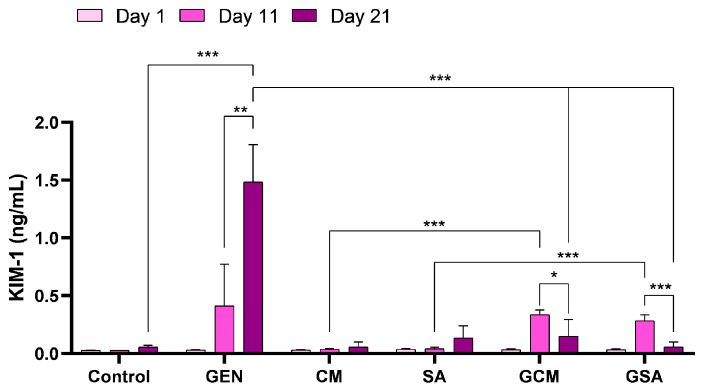
KIM-1 measurements in the experimental groups. Statistics performed using one-way ANOVA and two-way ANOVA; * *p* < 0.05, ** *p* < 0.01, *** *p* < 0.001. The outcomes represent mean ± SD of triplicate analyses.

As with the activity of CysC, the evaluation of KIM-1 was carried out employing the same ELISA method. As such, KIM-1 showed significantly increased readings in the antibiotic-treated rats compared to the untreated individuals (1.48 ± 0.32 vs. 0.05 ± 0.01 ng/mL, *p* < 0.001) on the 21st day of the experiment. Additionally, a substantial elevation in its levels was detected from one monitoring to another (*p* < 0.01), increasing from 0.03 ± 0.004 ng/mL on day 1 to 0.41 ± 0.35 ng/mL on day 11, and to the abovementioned reading on day 21. Simultaneously, significant reductions were detected in the GCM (0.15 ± 0.14 ng/mL) and GSA (0.05 ± 0.04 ng/mL) groups when compared to the GEN group (*p* < 0.001). KIM-1 readings within these groups significantly dropped between days 11 and 21 (0.33 ± 0.04 vs. 0.15 ± 0.14 ng/mL for the GCM group and 0.28 ± 0.04 vs. 0.05 ± 0.04 ng/mL for the GSA group, respectively, *p* < 0.001). Comparing the extract-treated batches, on day 11, a significant difference was observed in both the GCM (0.03 ± 0.008 vs. 0.33 ± 0.04 ng/mL, *p* < 0.001) and the GSA (0.04 ± 0.01 vs. 0.28 ± 0.04 ng/mL, *p* < 0.001) groups, whereas on day 21, these values were comparable to those found in the CM and SA groups (*p* > 0.05), outlining the protective effect of both extracts in gentamicin toxicity. Figure 5 displays a comprehensive reproduction of these results.

### 3.6. Urinary N-Acetyl-β-D-Glucosaminidase Index (iNAG) Activity Results

**Figure 6:**
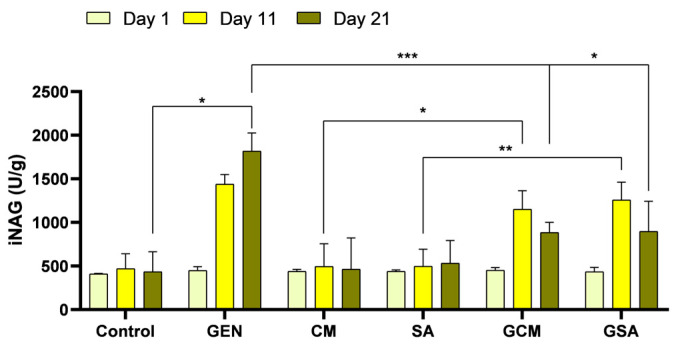
iNAG measurements in the experimental groups. Statistics performed utilizing one-way ANOVA and two-way ANOVA; * *p* < 0.05, ** *p* < 0.01, *** *p* < 0.001. The results are expressed as mean ± SD of three replicate analyses.

Following the assessment of urinary *N*-Acetyl-*β*-D-Glucosaminidase and urinary creatinine, as well as using the calculation formula described in the Materials and Methods section, the NAG index results were obtained and are revealed in Figure 6. As a result, on the trial’s last day, the gentamicin-treated rats’ NAG index activity was found to be significantly higher than the one registered in the negative control group (1817.18 ± 209.43 vs. 434.78 ± 229.43 U/g, *p* < 0.05). Importantly, by comparing the antibiotic and extract-treated groups with the gentamicin-treated subjects, a significant decrease in iNAG levels was registered, with extracts showing a protective effect at the renal tubular level (vs. 885.01 ± 116.88 U/g for the GCM group, *p* being < 0.001, and vs. 896.85 ± 344.92 U/g for the GSA group, *p* being < 0.05). Moreover, a significant difference in iNAG levels between the GCM and GSA groups and the groups that received only the extract was registered on day 11 (1151.78 ± 212.70 vs. 495.67 ± 259.62 U/g, *p* < 0.05, and 1258.04 ± 203.65 vs. 497.05 ± 195.43 U/g, respectively, *p* < 0.01), which decreased until day 21, when no significant difference was seen between these groups (*p* > 0.05). Thus, extracts provided to the GCM and GSA groups demonstrated a similar protective effect throughout time as in the CM and SA groups.

### 3.7. Nitro-Oxidative Stress Markers Activity Results

**Figure 7:**
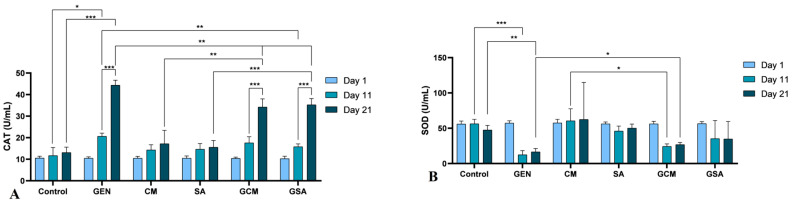

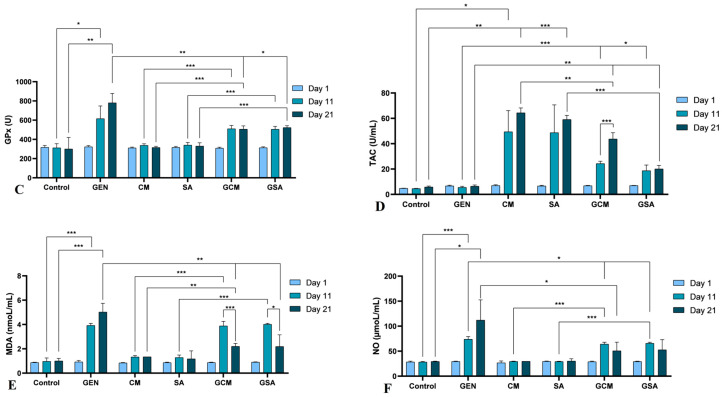
Nitro-oxidative stress profiling from plasma. (**A**) Catalase. (**B**) Superoxide dismutase. (**C**) Glutathione peroxidase. (**D**) Total antioxidant capacity. (**E**) Malondialdehyde. (**F**) Nitric oxide. Statistics performed utilizing one-way ANOVA and two-way ANOVA; * *p* < 0.05, ** *p* < 0.01, *** *p* < 0.001. The outcomes represent mean ± SD of three replicate assessments.

The assessment of certain parameters from plasma (CAT, SOD, GPx, TAC, MDA, NO) and tissues (total proteins, CAT, SOD, GPx, TAC, MDA, NO) made up the nitro-oxidative stress profiling in this study. Figure 7 displays the activity values of the markers derived from the plasma of the individuals under investigation.

As such, catalase (CAT) registered significantly increased activity in the gentamicin-treated group compared to the Control group on the 11th day (20.65 ± 1.45 vs. 11.70 ± 3.74 U/mL, *p* < 0.05), as well as on the 21st day of the trial (44.34 ± 2.32 vs. 13.11 ± 2.48 U/mL, *p* < 0.001). Additionally, CAT was observed to be increased from one monitoring to another (*p* < 0.001). Moreover, by comparing this group to the antibiotic and extract-treated rats, a significant reduction in CAT activity was registered in the GSA group on the 11th day of the experiment (15.80 ± 1.30 U/mL, *p* < 0.01), and also on the last day of the trial (35.26 ± 2.88 U/mL, *p* < 0.01). Regarding the GCM batch, no statistical difference was observed on the 11th day (17.58 ± 2.84 U/mL, *p* > 0.05), while on day 21, CAT activity was significantly decreased compared to the GEN-treated batch (34.21 ± 3.80 U/mL, *p* < 0.01). However, substantial rises were observed in both batches from one monitoring to another. (*p* < 0.001). Furthermore, by comparing these individuals to the extract only-treated rats, catalase activity was significantly elevated in both GCM (vs. 17.20 ± 6.21 U/mL, *p* < 0.01) and GSA (vs. 15.55 ± 3.12 U/mL, *p* < 0.001) groups on the 21st day of the trial. These results can be followed in Figure 7A.

Superoxide dismutase (SOD) recorded a significant decrease in its activity in the antibiotic-treated group in comparison to the Control group on both day 11 (12.42 ± 5.84 vs. 56.35 ± 6.17 U/mL, *p* < 0.001) and day 21 (16.61 ± 4.73 vs. 47.61 ± 6.27 U/mL, *p* < 0.01). Importantly, a significant increase in SOD activity was observed in the GCM group (26.87 ± 2.95 U/mL) on the 21st day of the experiment (*p* < 0.05). However, the GSA batch did not register any significant fluctuations in SOD levels in comparison to the gentamicin-treated group (*p* > 0.05). Moreover, by comparing the antibiotic and extract-treated rats to the extract only-treated rats, SOD recorded a significant decrease in its activity in the GCM group in comparison to the CM group on the 11th day (24.29 ± 3.50 vs. 60.57 ± 16.97 U/mL, *p* < 0.05); meanwhile, no significant difference was recorded on the 21st day between these experimental animals (*p* > 0.05). Regarding the *Sorbus aucuparia L.*-treated groups, SOD activity did not show any significant difference on the 11th day or on the last day of the trial (*p* > 0.05). Figure 7B displays these outcomes graphically.

In terms of glutathione peroxidase (GPx), a significant increase was observed in the GEN group compared to the Control group, starting from day 11 (615 ± 133.04 vs. 312 ± 43.27 U, *p* < 0.05), and continuing until day 21 (780 ± 97.98 vs. 300 ± 120.0 U, *p* < 0.01). Importantly, a significant reduction in GPx values was observed in both antibiotic and extract-treated groups on the 21st day of the experiment (vs. 506.5 ± 34.0 U, *p* being < 0.01, and vs. 522.5 ± 20.62 U, *p* being < 0.05). However, compared to the extract only-treated rats, significant differences were recorded on both day 11 and day 21 for both extracts (*p* < 0.001 vs. CM and SA). Figure 7C reveals a detailed reproduction of these results.

Regarding the total antioxidant capacity (TAC), the Control group exhibited no statistically significant difference from the gentamicin-treated group, with *p* > 0.05. Moreover, by comparing the negative control group to the extract only-treated groups, TAC levels in the CM group were considerably higher on day 11 (49.57 ± 16.61 vs. 4.58 ± 0.17 U/mL, *p* < 0.05), with an even greater rise on day 21 (vs. 64.33 ± 3.89, *p* < 0.01). Additionally, TAC values were significantly elevated in the SA group on the final day of the trial (59.11 ± 3.27 vs. 5.85 ± 0.7 U/mL, *p* < 0.001). Furthermore, statistical differences were seen on days 11 and 21 when comparing the gentamicin-treated group to the groups that had received antibiotic and extract treatment. As a result, the GCM group’s TAC values were significantly higher than those of the GEN group on days 11 (24.29 ± 1.86 vs. 5.53 ± 0.85 U/mL, *p* < 0.001) and 21 (43.70 ± 4.92 vs. 6.40 ± 1.02 U/mL, *p* < 0.01). Additionally, there was a substantial rise in TAC values between assessments (*p* < 0.001). Similar increases were recorded in the GSA group (18.7 ± 4.48 U/mL vs. control, with *p* < 0.05 and 20.10 ± 2.75 U/mL vs. control, with *p* < 0.01). These groups were then contrasted with the extract only-treated groups. As such, on day 11, there were no statistical differences (*p* > 0.05); however, on day 21, these values were significantly lower than those of the CM and SA groups (GCM vs. 64.33 ± 3.89 U/mL, *p* < 0.01, and GSA vs. 59.11 ± 3.27 U/mL, *p* < 0.001). These outcomes are displayed in detail in Figure 7D.

Malondialdehyde (MDA) increased significantly in the GEN group compared to the Control group on day 11 (3.92 ± 0.18 vs. 0.97 ± 0.28 nmoL/mL, *p* < 0.001), increasing further on day 21 (5.02 ± 0.72 vs. 1 ± 0.20 nmoL/mL, *p* < 0.001). An improvement in MDA values was observed in the GCM and GSA groups compared to the GEN group on the last day of the experiment (2.20 ± 0.24, 2.19 ± 0.96 nmoL/mL vs. GEN, *p* < 0.01), with the extracts showing a protective effect on the individuals. Moreover, MDA levels significantly decreased on day 21 compared to day 11 in both the GCM group (2.20 ± 0.24 vs. 3.88 ± 0.38 nmoL/mL, *p* < 0.001) and the GSA group (2.19 ± 0.96 vs. 4.02 ± 0.08 nmoL/mL, *p* < 0.05). However, several modifications were observed when contrasting these groups with the extract only-treated rats. Thus, on both days 11 (vs. 1.33 ± 0.12, nmoL/mL, *p* < 0.001) and 21 (vs. 1.34 ± 0.0 nmoL/mL, *p* < 0.01), the GCM group showed considerably higher values than the CM group. On the other hand, the GSA group had considerably higher values than the SA group on day 11 (vs. 1.28 ± 0.21 nmoL/mL, *p* < 0.001), but on day 21 these values were comparable to the extract only-treated group (vs. 1.18 ± 0.65 nmoL/mL, *p* > 0.05), suggesting a similar protective effect. Figure 7E depicts the sequence of these results.

Nitric oxide (NO) recorded a significantly increased activity in the GEN group compared to the Control group on both days 11 (73.98 ± 5.02 vs. 28.20 ± 1.26 µmoL/mL, *p* < 0.001) and 21 (112.09 ± 40.44 vs. 29.35 ± 0.67 µmoL/mL, *p* < 0.05). Importantly, the GCM group improved in NO activity on day 11 (64.03 ± 3.75 µmoL/mL vs. GEN, *p* < 0.05) as well as on day 21 (50.70 ± 17.16 µmoL/mL vs. GEN, *p* < 0.05), whereas the GSA group improved only on day 11 (65.71 ± 2.07 µmoL/mL vs. GEN, *p* < 0.05). Furthermore, by comparing the GCM and GSA groups to the CM and SA batches, a statistically significant increase in NO activity was recorded on day 11 (GCM vs. 29.49 ± 0.63 µmoL/mL and GSA vs. 29.19 ± 0.7 µmoL/mL, *p* < 0.001), while these values were comparable on day 21 (*p* > 0.05), with extracts manifesting a similar protective effect. Figure 7F displays these results graphically.

**Figure 8:**
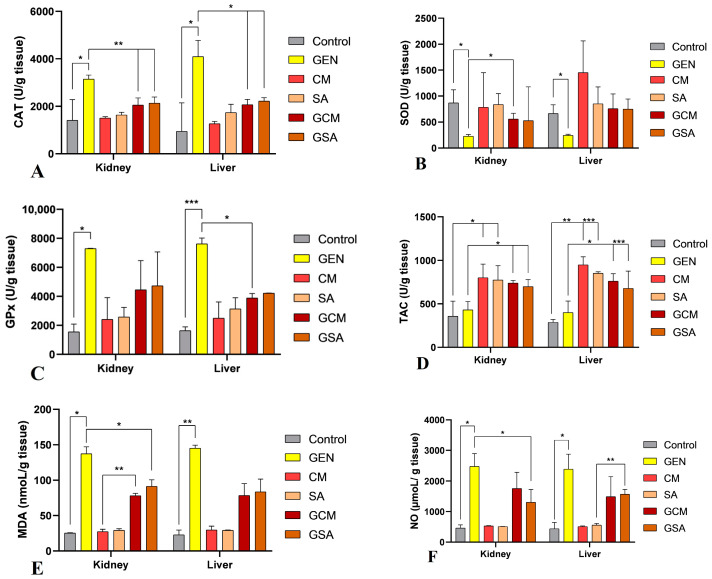
Nitro-oxidative stress profiling from tissue extracts. (**A**) Catalase. (**B**) Superoxide dismutase. (**C**) Glutathione peroxidase. (**D**) Total antioxidant capacity. (**E**) Malondialdehyde. (**F**) Nitric oxide. Statistics performed utilizing one-way ANOVA and two-way ANOVA; * *p* < 0.05, ** *p* < 0.01, *** *p* < 0.001. The findings represent mean ± SD of three replicate assessments.

Regarding tissue analyses, the activity values of the markers obtained from tissue homogenates of the subjects under evaluation are shown in Table 1 and Figure 8. The total protein content values were utilized to calculate and obtain the results of nitro-oxidative stress markers from tissue extracts.

As a result, CAT activity was significantly increased in the GEN group compared to the Control group, in both kidney (3145.30 ± 172.5 vs. 1410.35 ± 870.45 U/g, *p* < 0.05) and liver tissue (4098.16 ± 681.73 vs. 946.47 ± 1203.29 U/g, *p* < 0.05). A statistically significant decrease in CAT activity was detected in the experimental animals that received both an antibiotic and extract treatment in the kidney (2053.55 ± 298.86 and 2138.04 ± 252.29 U/g vs. GEN, *p* < 0.01) as well as in the liver tissue (2068.67 ± 223.17 and 2224.07 ± 138.17 U/g vs. GEN, *p* < 0.05). Importantly, no statistical difference was seen when comparing these groups to the extract only-treated rats in both tissues, indicating that the extracts modulate the antioxidant enzyme in a similar manner (*p* > 0.05). These results are generally consistent with the plasma outcomes and can be followed graphically in Figure 8A.

Regarding SOD activity, a statistically significant decrease was observed in the GEN group in comparison to the Control group in the kidney (228.04 ± 32.16 vs. 866.66 ± 253.22 U/g, *p* < 0.05) and liver tissue (244.13 ± 20.63 vs. 662.97 ± 170.23 U/g, *p* < 0.05). By comparing the antibiotic and extract-treated groups to the GEN group, a significant elevation in SOD activity was observed in the GCM group at the renal level (556.29 ± 111.99 U/g vs. GEN, *p* < 0.05). Simultaneously, an increase was seen in the GSA group, but without statistical significance (*p* > 0.05). In terms of liver tissue, increases in SOD levels were also observed in both batches, but no significant differences were recorded when compared to the GEN-treated rats (*p* > 0.05). Additionally, these results were comparable to those of the extract only-treated individuals in both tissues (*p* > 0.05). Figure 8B displays these data, which are essentially similar to the plasma results.

GPx registered a significantly increased activity in the GEN group compared to the Control group at the renal level (7295.62 ± 14.10 vs. 1551.61 ± 531.79 U/g, *p* < 0.05) as well as at the hepatic level (7617.63 ± 406.52 vs. 1641.0 ± 254.91 U/g, *p* < 0.001). Furthermore, in the kidney tissue, decreases in GPx activity were observed in the GCM and GSA groups, yet with no significant differences in comparison to the GEN group (*p* > 0.05). However, a significant reduction in GPx levels was observed in the GCM group at the hepatic level (3884.28 ± 310.55 U/g vs. GEN, *p* < 0.05). In addition, in both tissues, these outcomes were equivalent to those of the extract only-treated batches (*p* > 0.05). These results are partially consistent with the plasma analyses, and Figure 8C reveals these outcomes in detail.

In terms of TAC outcomes, similar to the modifications reported in plasma analysis, no significant differences were observed at the renal level between the Control group (357.57 ± 176.16 U/g) and the GEN group (430.82 ± 97.68 U/g), *p* being > 0.05. In contrast, significant increases in TAC levels were observed in the CM (802.15 ± 156.06 U/g) and SA (777.21 ± 162.65 U/g) groups in comparison to the saline-treated rats (*p* < 0.05). Subsequently, when comparing the GCM and GSA groups to the GEN group, a significant elevation in TAC levels was recorded, with extracts showing an antioxidant effect (741.23 ± 29.09 and 699.93 ± 80.14 U/g respectively, *p* < 0.05). Additionally, the protective effects shown in these batches were comparable to those observed in the extract only-treated groups (*p* > 0.05). Furthermore, similar modifications were seen in the liver tissue. Therefore, TAC levels were significantly low in the Control (287.52 ± 31.24 U/g) and GEN (399.04 ± 135.0 U/g) groups, but considerably greater in the CM (949.65 ± 92.60 U/g) and SA (853.54 ± 15.44 U/g) groups. Moreover, TAC increased significantly in the GCM and GSA groups compared to the GEN group (761.96 ± 86.26 U/g vs. GEN, *p* < 0.05, and 679.85 ± 196.90 U/g vs. GEN, *p* < 0.001). These levels were similar to those observed in the extract only-treated groups (*p* > 0.05). Figure 8D displays these outcomes graphically.

MDA levels in renal tissue increased significantly in the GEN group compared to the Control batch (137.46 ± 9.69 vs. 25.48 ± 0.45 nmoL/g, *p* < 0.05). Simultaneously, an improvement was observed in the GSA group (91.41 ± 9.12 nmoL/g) compared to the GEN group (*p* < 0.05), while the activity also decreased in the GCM group (78.13 ± 3.27 nmoL/g), but without statistical difference (*p* > 0.05). Additionally, a significant increase was seen in this group when compared to the extract only-treated subjects (vs. 27.36 ± 3.31 nmoL/g, *p* < 0.01). Furthermore, regarding liver MDA values, significant increases could be observed in the antibiotic-treated rats compared to the Control group (145.33 ± 4.04 vs. 22.85 ± 6.73 nmoL/g, *p* < 0.01). MDA decreased in the GCM and GSA groups compared to the GEN group, but without statistical significance (78.42 ± 16.79, 83.55 ± 18.02 nmoL/g vs. GEN, *p* > 0.05). Thus, by analyzing the results of MDA activity, it could be emphasized that *Cornus mas* L. and *Sorbus aucuparia* L. extracts exerted a stronger antioxidant effect at the plasma level than at the tissue level. Figure 8E depicts all of these variations.

NO significantly increased in its activity in the GEN group in comparison to the Control group in both kidney (2478.39 ± 417.5 vs. 465.23 ± 101.89 µmoL/g, *p* < 0.05) and liver tissue (2391.45 ± 485.599 vs. 441.9 ± 203.54 µmoL/g, *p* < 0.05). Importantly, a substantial reduction in NO levels was registered in the GSA group at the renal level (1304.34 ± 413.85 µmoL/g vs. GEN, *p* < 0.05). Additionally, a decrease was also observed in the GCM group, yet with no statistical significance (*p* > 0.05). Moreover, in the liver tissue, decreases in NO levels were registered in the GCM and GSA groups in comparison to the GEN group, but no statistical differences were observed (*p* > 0.05). As such, similar to MDA activity, both extracts manifest a greater antioxidant impact in plasma compared to tissue. Figure 8F reveals these outcomes graphically.

### 3.8. Histopathological Analysis

**Figure 9:**
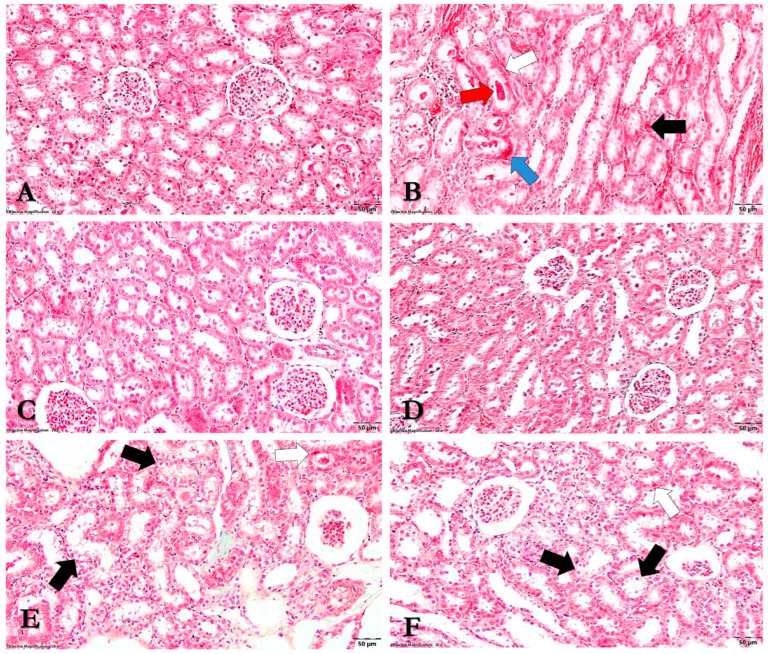
Histopathological analysis of the renal tissue. (**A**) Control group (rat no. 1, GT stain): normal aspect of the renal cortex; HS = 0. (**B**) Gentamicin group (rat no. 1, GT stain): septal congestion in the corticomedullary junction (black arrow), coagulative necrosis of the tubular epithelium (white arrow), and proteinaceous material in the lumen of the renal tubules (red arrow); scattered apoptotic cells in the epithelium lining uriniferous tubules (blue arrow); HS = 4. (**C**) *Cornus mas* L. group (rat no. 2, GT stain): normal features of the renal cortex; HS = 0. (**D**) *Sorbus aucuparia* L. group (rat no. 2, GT stain): unaltered histological aspect of the renal cortex; HS = 0. (**E**) Gentamicin + *Cornus mas* L. group (rat no. 4, GT stain): focal vacuolation of the tubular epithelium in the internal area of the renal cortex (black arrows) and isolated apoptotic cells of the tubular epithelium from the corticomedullary junction (white arrow); HS = 2. (**F**) Gentamicin + *Sorbus aucuparia* L. group (rat no. 5, GT stain): focal vacuolation of the tubular epithelium in the renal cortex (black arrows) and isolated apoptotic cells in the epithelium lining proximal and distal renal tubules (white arrow); HS = 2. Goldner’s trichrome stain—GT. Histopathological score—HS.

**Figure 10:**
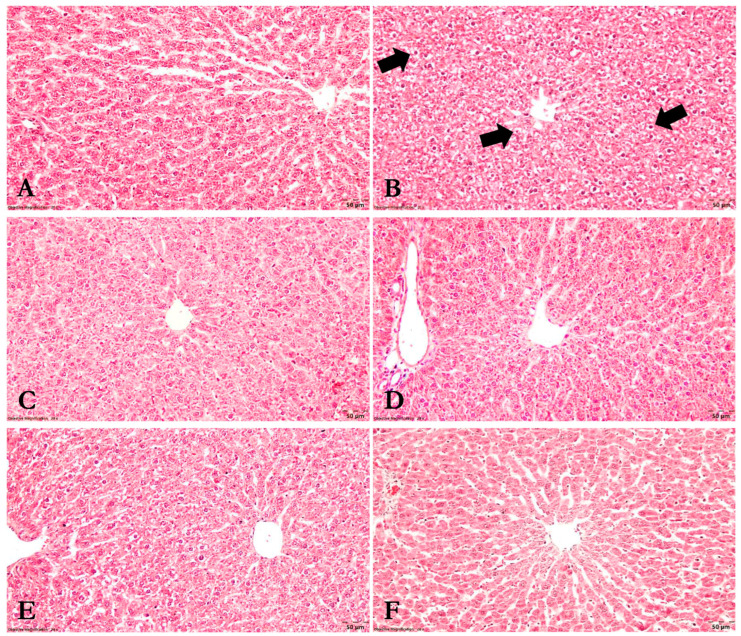
Histopathological features of the hepatic parenchyma in experimental groups. (**A**) Control group (rat no. 1, GT stain): normal histological aspect of the liver; HS = 0. (**B**) Gentamicin group (rat no. 3, GT stain): diffuse vacuolation of the hepatocytes (hepatosis) throughout the liver lobule (black arrows); HS = 3. (**C**) *Cornus mas* L. group (rat no. 1, GT stain): typical histological features of the liver in this group; HS = 0. (**D**) *Sorbus aucuparia* L. group (rat 3, GT stain): unchanged hepatic parenchyma; HS = 0. (**E**) Gentamicin + *Cornus mas* L. group (rat no. 4, GT stain): no alteration of the liver in this group; HS = 0. (**F**) Gentamicin + *Sorbus aucuparia* L. group (rat no. 6, GT stain): normal aspect of the liver; HS = 0. Trichrome Goldner stain. HS = Histopathological score.

The histopathological score defined in the Materials and Methods section was used to classify the histopathological modifications within each experimental group. The results were analyzed and are revealed in Table 2 and Figure 9 and Figure 10.

As such, the Control group showed no evident histological alterations, with adequate kidney and liver morphology (SH = 0). Gentamicin administration, on the other hand, caused multifocal vacuolation of the tubular epithelium in the renal cortex and medulla, coagulative necrosis, and the presence of proteinaceous material in the lumen of renal tubules. It also resulted in widespread septal congestion in the cortex and corticomedullary junction. Additionally, apoptotic cells in the tubular epithelium were also identified. The histopathological score in this group varied from 3 to 4, with a mean of 3.5 ± 0.57. This score was considerably higher (*p* < 0.01) when compared to the Control group’s score. Furthermore, diffuse hepatosis with hepatocyte vacuolation across the liver lobule structure was discovered at the hepatic level. Simultaneously, isolated hepatocyte apoptosis was also detected. This group received a histological liver score of 3, with the liver being severely impaired compared to the Control group (*p* < 0.001).

Moreover, after administering *Cornus mas* L. and *Sorbus aucuparia* L. extracts, no histological abnormalities were observed in either group, with appropriate kidney and liver morphology (SH = 0). Additionally, there were no significant changes between the extract-treated and Control groups (*p* > 0.05).

In the gentamicin and *Cornus mas* L. extract-treated group, focal vacuolation of the renal tubules, isolated apoptosis, and zonal septal congestion in the internal area of the renal cortex were observed. Areas of unmodified-appearing renal parenchyma were also identified. Within this group, the histopathological score ranged from 0 to 2, with a mean of 1.0 ± 1.0. Compared to the GEN group, there was a significant improvement in renal lesions, with *Cornus mas* L. extract showing a protective effect in renal tissue (*p* < 0.05). However, kidney impairment was still detected when compared to the group treated with extract alone, but without statistical significance (*p* > 0.05). Additionally, no histological abnormalities were found at the hepatic level, yielding an SH of 0 (*p* < 0.001 vs. GEN; *p* > 0.05 vs. CM).

Gentamicin and *Sorbus aucuparia* L. extract treatment resulted in focal vacuolar degenerative lesions and localized apoptosis in the tubular epithelium of the renal cortex, with much of the renal parenchyma appearing unmodified. The kidney histopathology score was calculated to be 0.66 ± 1.15. Thus, there were statistically significant improvements in kidney damage in this group compared to the GEN group (*p* < 0.05), and statistically insignificant lesions compared to the SA group (*p* > 0.05). The liver histopathology score was set to zero, indicating that the liver exhibited an ordinary histological appearance (*p* < 0.001 vs. GEN; *p* > 0.05 vs. SA). In brief, the *Sorbus aucuparia* L. extract exhibits a protective effect on both tissues in a similar manner to the *Cornus mas* L. extract.

## 4. Discussions

### 4.1. The Effects of Cornus mas L. and Sorbus aucuparia L. Extracts on Body Mass

The results concerning body weight measurements revealed that the gentamicin-treated rats showed dramatic declines in body mass. The antibiotic and extract-treated animals did not demonstrate significant weight loss, with herbs showing a beneficial impact on the body in comparison to the detrimental effects of gentamicin. According to an earlier study [42], gentamicin drastically reduced the body weight of rats, whereas oral treatment of gymnemic acid greatly recovered the body weight, which is in agreement with our findings. Another study [43] reported through weekly body mass monitoring that all rats gained weight continuously, demonstrating minor fluctuations in body weight during the research period. These variations could be attributed to the use of a different gentamicin dose, the environmental climate, and the diet provided.

### 4.2. The Effects of Cornus mas L. and Sorbus aucuparia L. Extracts on Hematological Parameters

The hematological profile revealed no statistically significant alterations. Our results were compared to data from the literature about other medicinal plants with potential nephroprotective effects. As such, in earlier in vivo nephrotoxicity trials, small variations in some hematological parameters were reported regardless of the treatment received [43,44]. Therefore, it can be considered that no substantial hematological changes occur in case of nephrotoxicity, and further research of other parameters is required.

### 4.3. The Effects of Cornus mas L. and Sorbus aucuparia L. Extracts on Total Proteins, Albumin, Globulins and Glucose

Total proteins increased in the gentamicin-treated group, whereas both extracts reduced their levels. The concentration of albumin, globulins, and glucose, on the other hand, was unaffected by either therapy. According to the literature’s data, decreases in total proteins and albumin following nephrotoxic substance treatment were reported, with *Cornus mas* L. extract reversing these changes [45]. This may be related to protein loss through the glomerular filtrate, whereas our study’s increase in total protein values could be attributed to dehydration. Furthermore, another study reported elevations in proteins after gentamicin administration, as the antioxidant therapy managed to diminish their levels [46]. Our results are in agreement with this previous investigation.

### 4.4. The Effects of Cornus mas L. and Sorbus aucuparia L. Extracts on Liver Function

Gentamicin treatment significantly elevated ALT and ALP enzymes, whereas *Cornus mas* L. and *Sorbus aucuparia* L. extract treatment alleviated these increases. Additionally, *Sorbus aucuparia* L. extract showed a stronger hepatoprotective effect than *Cornus mas* L. extract. Contrary to our study, Mesgari Abbasi et al. [23] reported decreases in ALT and ALP enzymes in rats treated with the toxic substance. Another study registered increases in ALT and ALP enzymes after gentamicin administration; antioxidant therapy managed to diminish ALT levels, yet did not decrease ALP enzyme [46]. The enzymes’ elevations in the gentamicin-treated batch in our investigation could be related to the establishment of liver distress, with *Cornus mas* L. and *Sorbus aucuparia* L. extracts manifesting a stronger hepatoprotective effect by substantially reducing both enzymes.

### 4.5. The Effects of Cornus mas L. and Sorbus aucuparia L. Extracts on Renal Function

The results regarding conventional renal biomarkers showed drastic increases in BUN and creatinine levels in the gentamicin group. Following administration of both extracts, decreases in BUN levels were noted, but were not statistically significant. On the other hand, creatinine concentration substantially diminished after herbal treatment. To our current knowledge, the protective effect of *Cornus mas* L. extract in drug-induced nephrotoxicity in vivo has only been investigated in two previous research studies. Thus, Es Haghi et al. [45] reported increases in BUN and creatinine values following carbon tetrachloride treatment, whereas the *Cornus mas* L. extract alleviated these changes. Another study showed significant elevations in renal biomarkers, which were reduced through the use of *Cornus mas* L. extract [47]. Moreover, due to the fact that this is the first study to our knowledge that investigates the protective advantages of *Sorbus aucuparia* L. extract in an in vivo nephrotoxicity trial, the renal biomarkers were contrasted to those of other herbal components with possible nephroprotective qualities. As such, substantial elevations in BUN and creatinine readings were noted following gentamicin treatment, whereas herbal therapy succeeded in reducing their concentration [46,48]. The non-significant reduction in BUN levels in the antibiotic and extracts-treated group in our study could be related to the necessity of providing the extracts over a longer length of time or at a larger dosage, as there is already an improvement in nephrotoxicity.

Cystatin C is a protein with a low molecular weight that is constantly synthesized by all nucleated cells, freely removed by the renal glomeruli, and metabolized in the renal proximal tubules. CysC appears to be impacted by fewer non-glomerular filtration rate (GFR) elements and is less extensive than creatinine [49]. Although creatinine has been utilized for assessing GFR since the latter half of the 1950s, multiple studies suggest that CysC concentration could be employed as a substitute for creatinine to diagnose early GFR dysfunction [50]. As such, the results regarding CysC activity in our study revealed significant increases in the gentamicin-treated group. The administration of extracts initially resulted in a significant decrease in its values; nevertheless, CysC levels eventually started to increase, being similar to those of the gentamicin batch. Al-Kuraishy et al. [51] and Abdelkader et al. [52] reported increases in CysC activity following gentamicin therapy, which were reversed by the administration of natural antioxidants, demonstrating protective benefits on renal glomeruli. Based on these findings, it is reasonable to conclude that extracts of *Cornus mas* L. and *Sorbus aucuparia* L. do not possess a curative effect, but rather a preventive impact at the renal glomerular level. Further research is required to determine the mechanism and site of action of these extracts.

KIM-1 is a transmembrane glycoprotein that is not physiologically present at the renal level; it is expressed in the proximal tubular epithelium during pathophysiological conditions or when exposed to nephrotoxic substances [53]. Lately, increased blood levels of KIM-1 have been linked to both acute and chronic kidney injury. This biomarker has proven to be a remarkable indicator of renal damage in rat experimental models, surpassing BUN and creatinine as predictors of histopathological changes in the proximal tubules [54]. In the current study, KIM-1 levels significantly increased in the gentamicin-treated rats due to the occurrence of renal impairment, whereas administration of both extracts substantially decreased its levels. These results were further contrasted to those of other therapeutic plants that had previously been reported in the literature. As such, KIM-1 considerably increased after gentamicin injection, while pomegranate and garlic aqueous extract treatment drastically reduced the biomarker’s values [51,55]. Thus, it can be considered that our findings are in agreement with this previously published research and that both extracts demonstrated a protective effect against gentamicin nephrotoxicity by analysis of the activity of KIM-1.

NAG represents a lysosomal enzyme that is located primarily in the renal proximal tubular cells. NAG cannot be filtered via the typical glomerular basal membrane due to its high molecular weight. As its increase implies the damage of tubular cells, it serves as an accurate diagnostic for estimating tubular injury. Because the urinary NAG/creatinine ratio exhibits fewer fluctuations linked to urine volume or excretion rate than NAG levels alone, it is commonly presented in ratio to the urinary creatinine as urinary NAG index (iNAG) activity [56,57]. In our study, gentamicin therapy resulted in large increases in iNAG activity, while *Cornus mas* L. and *Sorbus aucuparia* L. extract treatment drastically decreased its activity levels, outlining the protective effect against gentamicin’s harmful effects. Because the effects of these extracts on iNAG activity in an experimental model of nephrotoxicity had not previously been studied, our findings were compared to those of other investigated components. Thus, Codea et al. [58] reported rises in iNAG activity in gentamicin-treated rats, whereas the administration of melatonin and erythropoietin prevented this aspect. Another study demonstrated the nephroprotective effect of *Ginkgo biloba* extract by determining significant reductions in iNAG levels in rats exposed to vancomycin-induced kidney damage [59]. Therefore, our results are in accordance with these previous publications, with *Cornus mas* L. and *Sorbus aucuparia* L. extracts manifesting a protective effect at the renal tubular level.

### 4.6. The Antioxidant Activity of Cornus mas L. and Sorbus aucuparia L. Extracts

In terms of the results of antioxidant enzymes, a variety of changes were noticed. Thus, catalase levels were greatly increased after receiving gentamicin therapy, whereas treatment with the two extracts significantly decreased its activity. Additionally, both extracts indicated a stronger antioxidant impact at the tissue level than in plasma. SOD activity drastically decreased after receiving gentamicin treatment. Its activity was significantly increased after treatment with *Cornus mas* L. extract, and insignificantly elevated following *Sorbus aucuparia* L. extract administration. Moreover, plasma-level GPx activity was seen to increase in the gentamicin-treated rats before considerably declining following antioxidant treatment. When compared to plasma, the enzyme activity at the tissue level was shown to be lower, yet manifested a stronger antioxidant activity in liver tissue. Further, the groups treated with antioxidants alone demonstrated the greatest increases in plasma TAC activity. The antibiotic-plus-extracts groups demonstrated an improvement in antioxidant capacity, but they still differed significantly from the extracts-only batches. At the tissue level, the extracts manifested a higher antioxidant capacity than in plasma. These statistics were compared to other findings that had been documented in the literature. Thus, in a previously mentioned study [45], carbon tetrachloride caused a decrease in CAT, SOD, and GPx activity, while the administration of *Cornus mas* L. extract improved the activity of these enzymes. The same results were observed in the study by Mesgari Abbasi et al. [23]. According to another study [46], the administration of cisplatin had no appreciable effects on the activity of antioxidant enzymes. However, TAC activity was considerably higher and GPx was markedly reduced in the cisplatin + *Cornus mas* L. groups. In other prior studies, the values of CAT, SOD, and GPx were markedly reduced following gentamicin administration, whereas the antioxidant therapy enhanced their activity [60,61]. Tomşa et al. [62] discovered TAC values to be substantially reduced after gentamicin treatment, whereas the administration of curcumin and vitamin C determined a rise in its levels.

Our investigation supports earlier publications’ findings regarding the activity of the SOD enzyme. In contrast, the enhanced CAT and GPx activity in the gentamicin group might be caused by the cellular reaction to intrinsic oxidative stress induced by the antibiotic. Regarding the increased TAC levels in the extracts group, it can be assumed that this marker is strongly connected to the exogenous intake of antioxidants through diet, so a high value of this parameter may be explained as either a reduced ROS production or as an indicator of antioxidant dietary consumption [63].

### 4.7. The Effects of Cornus mas L. and Sorbus aucuparia L. Extracts on Nitro-Oxidative Markers

Malondialdehyde represents a trustworthy parameter of oxidative stress that is a byproduct of lipid peroxidation, which denotes an increased formation of free radicals [60]. In our study, MDA activity measurements revealed a considerable rise in the gentamicin therapy group. Following treatment with both extracts, these elevations were reduced, demonstrating a potential antioxidant impact. However, the capacity of the extracts to reduce MDA levels in tissues was reduced as compared to plasma activity. According to the literature’s data, the administration of nephrotoxic substances determined an increase in MDA activity, while the treatment with *Cornus mas* L. extract diminished the production of ROS [23,45]. Additionally, other studies demonstrated an elevation in MDA levels following gentamicin administration, whereas the antioxidant-based treatment alleviated lipid peroxidation [5,43,48]. The current paper’s findings regarding *Cornus mas* L. and *Sorbus aucuparia* L. fruits’ impact on malondialdehyde activity are in agreement with these earlier publications.

Nitric oxide is believed to play an important part in renal physiopathological mechanisms. The alteration of renal hemodynamics and tubular function, as well as the disruption in NO’s synthesis pathway, have all been linked to kidney dysfunction [64]. As such, a sudden rise in the concentration of nitric oxide produced by nitric oxide synthase (NOS), in the presence of high quantities of superoxide anions (O_2_^−^), generates the formation of reactive nitrogen species (RNS), specifically peroxynitrite, which determines cytotoxic effects and ultimately cell death [65,66]. In our study, a substantial rise in NO levels was seen after gentamicin administration, in both plasma and tissues. Following antioxidant treatment, *Cornus mas* L. extract demonstrated a stronger antioxidant effect in plasma, while *Sorbus aucuparia* L. extract showed a more pronounced antioxidant action at the renal tissue level. Moreover, previous investigations indicated increases in NO levels after gentamicin treatment, which were subsequently decreased by antioxidant therapy through the reduction of NOS and oxidative stress [5,48,63]. Our findings are broadly in agreement with this prior research. The delivery of gentamicin may have caused oxidative stress, which in turn caused NO to be scavenged by superoxide and peroxynitrite to be produced. The resulting harm to the tubular cells may have caused renal malfunction. Additionally, the positive association discovered between NO and MDA in our study emphasizes this assertion, implying that higher NO is related to high levels of oxidative stress, as the antioxidant therapy ameliorates the production of ROS and RNS.

### 4.8. The Effects of Cornus mas L. and Sorbus aucuparia L. Extracts on Kidney and Liver Tissue

According to the results of the histopathological analysis, the animals that received gentamicin experienced severe alterations in the kidney and liver tissue. Significant changes were observed after extracts treatment, with the majority of the renal and hepatic parenchyma appearing unaltered. Even so, as compared to the groups that received extracts alone, kidney damage was still observed, although without statistically significant differences. According to previously published data, the administration of carbon tetrachloride and cisplatin caused glomerular and tubular lesions, as well as hepatic alterations. *Cornus mas* L. extract treatment improved these changes by reducing the severity of the lesions [23,45,46]. Other studies demonstrated that gentamicin injection led to renal and hepatic histological alterations, whereas gymnemic acid, *Cinnamomum zeylanicum* extract, and pycnogenol alleviated these changes [42,44,67]. In essence, it may be said that our results substantially support these previously released data.

**Figure 11:**
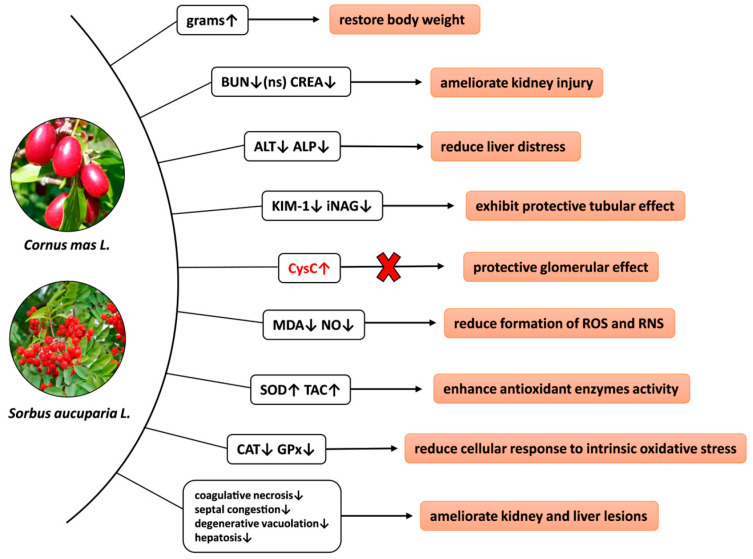
Mechanistic overview of the *Cornus mas* L. and *Sorbus aucuparia* L. -mediated modulation of gentamicin-induced nephrotoxicity; non-significant—ns; CysC levels were significantly increased outlining that the extracts did not determine a protective glomerular impact—red font.

In summary, Figure 11 illustrates a schematic representation of all impacts of *Cornus mas* L. and *Sorbus aucuparia* L. extracts on rats exposed to gentamicin stress.

## 5. Conclusions

*Cornus mas* L. and *Sorbus aucuparia* L. fruits have been shown to possess nephroprotective effects in gentamicin-induced nephrotoxicity on rats. Therefore, due to their ability to reduce oxidative and nitrosative stress as well as the particular renal biomarkers KIM-1 and iNAG, both extracts have demonstrated outstanding nephroprotection and a significant tubular protective effect. These findings were strengthened by histopathological changes and biochemical analysis. Moreover, the specific renal biomarker CysC did not show a substantial reduction in its levels, indicating that the extracts did not demonstrate a strong protective impact at the renal glomerular level. Further research is needed to establish the underlying process and active site of these fruits.

## Figures and Tables

**Table 1:** Total protein content evaluation of tissue homogenates.

Experimental Groups	Total Proteins (mg/g Tissue)	
	Kidney	Liver
Control	27.15 ± 4.13	25.18 ± 3.63
Gentamicin	42.87 ± 23.19	78.84 ± 21.95
*Cornus mas* L.	22.57 ± 4.18	40.50 ± 20.67
*Sorbus aucuparia* L.	28.82 ± 4.29	38.66 ± 4.75
Gentamicin + *Cornus mas* L.	40.87 ± 17.03	59.53 ± 15.28
Gentamicin + *Sorbus aucuparia* L.	31.93 ± 8.58	44.22 ± 7.59

**Table 2:** Histopathological score obtained from microscopic analysis of kidney and liver sections.

Experimental Groups	Histopathological Score (HS)			
	Kidney		Liver	
	Mean ± S.D	Range	Mean ± S.D	Range
Control	0	0	0	0
Gentamicin	3.5 ± 0.57 ^a^	3–4	3 ^e^	3
*Cornus mas* L.	0	0	0	0
*Sorbus aucuparia* L.	0	0	0	0
Gentamicin + *Cornus mas* L.	1.0 ± 1.0 ^b,c^	0–2	0 ^f^	0
Gentamicin + *Sorbus aucuparia* L.	0.66 ± 1.15 ^b,d^	0–2	0 ^f^	0

Statistics performed utilizing one-way ANOVA; ^a^ *p* < 0.01 vs. Control; ^b^ *p* < 0.05 vs. GEN; ^c^ *p* > 0.05 vs. CM; ^d^ *p* > 0.05 vs. SA; ^e^ *p* < 0.001 vs. Control; ^f^ *p* < 0.001 vs. GEN.

## Data Availability

The data presented in the study are available in the article.

## References

[1] Kwiatkowska E., Domański L., Dziedziejko V., Kajdy A., Stefańska K., Kwiatkowski S. (2021). The Mechanism of Drug Nephrotoxicity and the Methods for Preventing Kidney Damage. Int. J. Mol. Sci..

[2] Gameiro J., Fonseca J.A., Outerelo C., Lopes J.A. (2020). Acute Kidney Injury: From Diagnosis to Prevention and Treatment Strategies. J. Clin. Med..

[3] Legatti S.A.M., El Dib R., Legatti E., Botan A.G., Camargo S.E.A., Agarwal A., Barretti P., Paes A.C. (2018). Acute kidney injury in cats and dogs: A proportional meta-analysis of case series studies. PLoS ONE.

[4] Hoste E.A.J., Kellum J.A., Selby N.M., Zarbock A., Palevsky P.M., Bagshaw S.M., Goldstein S.L., Cerdá J., Chawla L.S. (2018). Global epidemiology and outcomes of acute kidney injury. Nat. Rev. Nephrol..

[5] Sarwar S., Hossain M.J., Irfan N.M., Ahsan T., Arefin M.S., Rahman A., Alsubaie A., Alharthi B., Khandaker M.U., Bradley D.A. (2022). Renoprotection of Selected Antioxidant-Rich Foods (Water Spinach and Red Grape) and Probiotics in Gentamicin-Induced Nephrotoxicity and Oxidative Stress in Rats. Life.

[6] Mody H., Ramakrishnan V., Chaar M., Lezeau J., Rump A., Taha K., Lesko L., Ait-Oudhia S. (2020). A Review on Drug-Induced Nephrotoxicity: Pathophysiological Mechanisms, Drug Classes, Clinical Management, and Recent Advances in Mathematical Modeling and Simulation Approaches. Clin. Pharmacol. Drug Dev..

[7] Bird L., Walker D. (2015). Pathophysiology of acute kidney injury. Companion Anim..

[8] Petejova N., Martinek A., Zadrazil J., Kanova M., Klementa V., Sigutova R., Kacirova I., Hrabovsky V., Svagera Z., Stejskal D. (2020). Acute Kidney Injury in Septic Patients Treated by Selected Nephrotoxic Antibiotic Agents—Pathophysiology and Biomarkers—A Review. Int. J. Mol. Sci..

[9] Balakumar P., Rohilla A., Thangathirupathi A. (2010). Gentamicin-induced nephrotoxicity: Do we have a promising therapeutic approach to blunt it?. Pharmacol. Res..

[10] Bencheikh N., Bouhrim M., Kharchoufa L., Al Kamaly O.M., Mechchate H., Es-Safi I., Dahmani A., Ouahhoud S., El Assri S., Eto B. (2021). The Nephroprotective Effect of *Zizyphus lotus* L. (Desf.) Fruits in a Gentamicin-Induced Acute Kidney Injury Model in Rats: A Biochemical and Histopathological Investigation. Molecules.

[11] Quiros Y., Vicente-Vicente L., Morales A.I., López-Novoa J.M., López-Hernández F.J. (2011). An Integrative Overview on the Mechanisms Underlying the Renal Tubular Cytotoxicity of Gentamicin. Toxicol. Sci..

[12] Randjelovic P., Veljkovic S., Stojiljkovic N., Sokolovic D., Ilic I. (2017). Gentamicin nephrotoxicity in animals: Current knowledge and future perspectives. Excli J..

[13] Bayram H.M., Ozturkcan S.A. (2020). Bioactive components and biological properties of cornelian cherry (*Cornus mas* L.): A comprehensive review. J. Funct. Foods.

[14] Tiptiri-Kourpeti A., Fitsiou E., Spyridopoulou K., Vasileiadis S., Iliopoulos C., Galanis A., Vekiari S., Pappa A., Chlichlia K. (2019). Evaluation of Antioxidant and Antiproliferative Properties of *Cornus mas* L. Fruit Juice. Antioxidants.

[15] Dinda B., Kyriakopoulos A.M., Dinda S., Zoumpourlis V., Thomaidis N.S., Velegraki A., Markopoulos C., Dinda M. (2016). *Cornus mas* L. (cornelian cherry), an important European and Asian traditional food and medicine: Ethnomedicine, phytochemistry and pharmacology for its commercial utilization in drug industry. J. Ethnopharmacol..

[16] Hosseinpour-Jaghdani F., Shomali T., Gholipour-Shahraki S., Rahimi-Madiseh M., Rafieian-Kopaei M. (2017). *Cornus mas*: A review on traditional uses and pharmacological properties. J. Complement. Integr. Med..

[17] Szczepaniak O.M., Kobus-Cisowska J., Kusek W., Przeor M. (2019). Functional properties of Cornelian cherry (*Cornus mas* L.): A comprehensive review. Eur. Food Res. Technol..

[18] Tenuta M.C., Deguin B., Loizzo M.R., Cuyamendous C., Bonesi M., Sicari V., Trabalzini L., Mitaine-Offer A.-C., Xiao J., Tundis R. (2022). An Overview of Traditional Uses, Phytochemical Compositions and Biological Activities of Edible Fruits of European and Asian Cornus Species. Foods.

[19] Dzydzan O., Brodyak I., Strugała-Danak P., Strach A., Kucharska A.Z., Gabrielska J., Sybirna N. (2022). Biological Activity of Extracts of Red and Yellow Fruits of *Cornus mas* L.—An In Vitro Evaluation of Antioxidant Activity, Inhibitory Activity against α-Glucosidase, Acetylcholinesterase, and Binding Capacity to Human Serum Albumin. Molecules.

[20] Antolak H., Czyzowska A., Sakač M., Mišan A., Đuragić O., Kregiel D. (2017). Phenolic Compounds Contained in Little-known Wild Fruits as Antiadhesive Agents Against the Beverage-Spoiling Bacteria *Asaia* spp. Molecules.

[21] Cosmulescu S.N., Trandafir I., Cornescu F. (2018). Antioxidant Capacity, Total Phenols, Total Flavonoids and Colour Component of Cornelian Cherry (*Cornus mas* L.) Wild Genotypes. Not. Bot. Horti Agrobot..

[22] Yılmaz S., Göçmen A.Y., Karataş E., Tokpınar A. (2020). *Cornus mas* L improves Antioxidant Status in the Liver, Lung, Kidney, Testis and Brain of Ehrlich Ascites Tumor Bearing Mice. Asian Pac. J. Cancer Prev..

[23] Abbasi M.M., Hassanalilou T., Khordadmehr M., Vardin A.M., Kohlan A.B., Khalili L. (2020). Effects of *Cornus mas* Fruit Hydro-Methanolic Extract on Liver Antioxidants and Histopathologic Changes Induced by Cisplatin in Rats. Indian J. Clin. Biochem..

[24] Dzydzan O., Bila I., Kucharska A.Z., Brodyak I., Sybirna N. (2019). Antidiabetic effects of extracts of red and yellow fruits of cornelian cherries (*Cornus mas* L.) on rats with streptozotocin-induced diabetes mellitus. Food Funct..

[25] Bobinaitė R., Grootaert C., Van Camp J., Šarkinas A., Liaudanskas M., Žvikas V., Viškelis P., Venskutonis P.R. (2020). Chemical composition, antioxidant, antimicrobial and antiproliferative activities of the extracts isolated from the pomace of rowanberry (*Sorbus aucuparia* L.). Food Res. Int..

[26] Rutkowska M., Kolodziejczyk-Czepas J., Olszewska M.A. (2021). The Effects of *Sorbus aucuparia* L. Fruit Extracts on Oxidative/Nitrative Modifications of Human Fibrinogen, Impact on Enzymatic Properties of Thrombin, and Hyaluronidase Activity In Vitro. Antioxidants.

[27] Cristea E., Ghendov-Mosanu A., Patras A., Socaciu C., Pintea A., Tudor C., Sturza R. (2021). The Influence of Temperature, Storage Conditions, pH, and Ionic Strength on the Antioxidant Activity and Color Parameters of Rowan Berry Extracts. Molecules.

[28] Rutkowska M., Kolodziejczyk-Czepas J., Owczarek A., Zakrzewska A., Magiera A., Olszewska M.A. (2021). Novel insight into biological activity and phytochemical composition of *Sorbus aucuparia* L. fruits: Fractionated extracts as inhibitors of protein glycation and oxidative/nitrative damage of human plasma components. Food Res. Int..

[29] Sarv V., Venskutonis P.R., Rätsep R., Aluvee A., Kazernavičiūtė R., Bhat R. (2021). Antioxidants Characterization of the Fruit, Juice, and Pomace of Sweet Rowanberry (*Sorbus aucuparia* L.) Cultivated in Estonia. Antioxidants.

[30] Aurori M., Niculae M., Hanganu D., Pall E., Cenariu M., Vodnar D.C., Bunea A., Fiţ N., Andrei S. (2023). Phytochemical Profile, Antioxidant, Antimicrobial and Cytoprotective Effects of Cornelian Cherry (*Cornus mas* L.) Fruit Extracts. Pharmaceuticals.

[31] Maurer L.H., Cazarin C.B.B., Quatrin A., Minuzzi N.M., Nichelle S.M., Lamas C.D.A., Cagnon V.H.A., Morari J., Velloso L.A., Maróstica M.R. (2020). Grape peel powder attenuates the inflammatory and oxidative response of experimental colitis in rats by modulating the NF-κB pathway and activity of antioxidant enzymes. Nutr. Res..

[32] Laczkó-Zöld E., Szabó D., Fogarasi E., Tömösközi-Farkas R., Ştefănescu R., Varga E., Eşianu S. (2018). Contribution to the Phytochemical Evaluation of Rowanberry Fruits (*Sorbus aucuparia* L.). Acta Medica Marisiensis.

[33] Dumitraş D.-A., Dreanca A.I., Pall E., Gal A.F., Rus V., Morohoschi A.G., Cotul M., Nan M.I., Andrei S. (2022). Inhibition of Tumor Growth and Modulation of Antioxidant Activity of Rhodoxanthin Isolated from Taxus baccata Aril against B16F10 Murine Malignant Melanoma. Antioxidants.

[34] Xue F., Li C., Pan S. (2013). In vivo antioxidant activity of carotenoid powder from tomato byproduct and its use as a source of carotenoids for egg-laying hens. Food Funct..

[35] Kini R.D., Arunkumar N., Gokul M. (2019). Potential Protective Role of Beta Carotene on Cadmium Induced Brain and Kidney Damage. Indian J. Public Health Res. Dev..

[36] (2006). Biological Evaluation of Medical Devices—Part 2: Animal Welfare Requirements.

[37] AMVA Panel of Euthanasia (2020). Amva Guidelines for the Euthanasia of Animals: 2020 Edition.

[38] Codea A.R., Mircean M., Sarpataki O., Sevastre B., Giurgiu G., Popovici C., Scurtu I., Neagu D., Oana L. (2018). Urinary N-Acetyl-Beta-D-Glucosaminidase Index Activity Normal Values in Healthy Wistar Rats. Bull. Univ. Agric. Sci. Vet. Med. Cluj-Napoca Vet. Med..

[39] Evelson P., Travacio M., Repetto M., Escobar J., Llesuy S., Lissi E.A. (2001). Evaluation of Total Reactive Antioxidant Potential (TRAP) of Tissue Homogenates and Their Cytosols. Arch. Biochem. Biophys..

[40] Weichselbaum C.T.E. (1946). An Accurate and Rapid Method for the Determination of Proteins in Small Amounts of Blood Serum and Plasma. Am. J. Clin. Pathol..

[41] Johnson-Delaney C.A. (1996). Exotic Companion Medicine Handbook for Veterinarians.

[42] Gumbar S., Bhardwaj S., Mehan S., Khan Z., Narula A.S., Kalfin R., Tabrez S., Zughaibi T.A., Wasi S. (2023). Renal mitochondrial restoration by gymnemic acid in gentamicin-mediated experimental nephrotoxicity: Evidence from serum, kidney and histopathological alterations. Front. Pharmacol..

[43] Rizwan F., Yesmine S., Banu S.G., Chowdhury I.A., Hasan R., Chatterjee T.K. (2019). Renoprotective effects of stevia (*Stevia rebaudiana* Bertoni), amlodipine, valsartan, and losartan in gentamycin-induced nephrotoxicity in the rat model: Biochemical, hematological and histological approaches. Toxicol. Rep..

[44] Atsamo A.D., Songmene A.L., Donfack M.F.M., Ngouateu O.B., Nguelefack T.B., Dimo T. (2021). Aqueous Extract from *Cinnamomum zeylanicum* (Lauraceae) Stem Bark Ameliorates Gentamicin-Induced Nephrotoxicity in Rats by Modulating Oxidative Stress and Inflammatory Markers. Evid. Based Complement. Altern. Med..

[45] Es Haghi M., Dehghan G., Banihabib N., Zare S., Mikaili P., Panahi F. (2014). Protective effects of *Cornus mas* fruit extract on carbon tetrachloride induced nephrotoxicity in rats. Indian J. Nephrol..

[46] Babaeenezhad E., Nouryazdan N., Nasri M., Ahmadvand H., Sarabi M.M. (2021). Cinnamic acid ameliorate gentamicin-induced liver dysfunctions and nephrotoxicity in rats through induction of antioxidant activities. Heliyon.

[47] Vardin A.M., Khordadmehr M., Heidari R., Nouri H.-O.-L., Amirkhiz M.B., Abbasi M.M. (2018). The Effects of *Cornus mas* Hydro-Methanolic Extract on Cisplatin-Induced Nephrotoxicity in Rats. Pharm. Sci..

[48] Edeogu C.O., Kalu M.E., Famurewa A.C., Asogwa N.T., Onyeji G.N., Ikpemo K.O. (2020). Nephroprotective Effect of Moringa Oleifera Seed Oil on Gentamicin-Induced Nephrotoxicity in Rats: Biochemical Evaluation of Antioxidant, Anti-inflammatory, and Antiapoptotic Pathways. J. Am. Coll. Nutr..

[49] Chen D.C., Potok O.A., Rifkin D., Estrella M.M. (2022). Advantages, Limitations, and Clinical Considerations in Using Cystatin C to Estimate GFR. Kidney360.

[50] Malmgren L., Öberg C., den Bakker E., Leion F., Siódmiak J., Åkesson A., Lindström V., Herou E., Dardashti A., Xhakollari L. (2023). The complexity of kidney disease and diagnosing it—Cystatin C, selective glomerular hypofiltration syndromes and proteome regulation. J. Intern. Med..

[51] Al-Kuraishy H.M., Al-Gareeb A.L.I.I., Al-Naimi M.S.S. (2019). Pomegranate attenuates acute gentamicin-induced nephrotoxicity in Sprague-Dawley rats: The potential antioxidant and anti-inflammatory effects. Pomegranate.

[52] Abdelkader R.S.E., El-Beih N.M., Zaahkouk S.A., El-Hussieny E.A. (2022). Ameliorative Effect of Eruca sativa seeds and its rutin on gentamicin-induced nephrotoxicity in male rats via targeting inflammatory status, oxidative stress and kidney injury molecule-1 (KIM-1)/cystatin c expression. Indones. Biomed. J..

[53] Jana S., Mitra P., Roy S. (2023). Proficient Novel Biomarkers Guide Early Detection of Acute Kidney Injury: A Review. Diseases.

[54] Brilland B., Boud’hors C., Wacrenier S., Blanchard S., Cayon J., Blanchet O., Piccoli G.B., Henry N., Djema A., Coindre J.-P. (2023). Kidney injury molecule 1 (KIM-1): A potential biomarker of acute kidney injury and tubulointerstitial injury in patients with ANCA-glomerulonephritis. Clin. Kidney J..

[55] Galal H.M., Abd el-Rady N.M. (2019). Aqueous garlic extract supresses experimental gentamicin induced renal pathophysiology mediated by oxidative stress, inflammation and Kim-1. Pathophysiology.

[56] Lee H.N., Jung J.Y., Hwang S., Park J.W., Kim D., Kwak Y.H., Lee B.J., Lee E.J. (2021). Role of the urinary N-acetyl-beta-D-glucosaminidase/creatinine (NAG/Cr) ratio in discriminating between true and false pyuria in sterile urine bag specimens. J. Pediatr. Urol..

[57] Tanaka S.I., Fujioka Y., Tsujino T., Ishida T., Hirata K.I. (2022). Association between urinary N-acetyl-β-glucosaminidase activity–urinary creatinine concentration ratio and risk of disability and all-cause mortality. PLoS ONE.

[58] Codea A.R., Mircean M., Nagy A., Sarpataky O., Sevastre B., Stan R.L., Hangan A.C., Popovici C., Neagu D., Purdoiu R. (2019). Melatonine and Erythropoietin Prevents Gentamicin Induced Nephrotoxicity in Rats. Farmacia.

[59] Popa R., Zaharie S.I., Diaconu M., Damian A., Varut M.C., Aurori M., Tabaran A.F., Constantinescu E., Codea A.R., Caragea D.C. (2021). Ginkgo biloba nephroprotective effects in animal models with vancomycin-induced nephrotoxicity. Rev. Romana Med. Vet..

[60] Bai R., Fan J., Wang Y., Wang Y., Li X., Hu F. (2023). Protective effect of Cistanche deserticola on gentamicin-induced nephrotoxicity in rats. Chin. Herb. Med..

[61] Léonard D.F., Ballo M., Soudre A., Tindano B., Bah S., Bayala B. (2023). Effects of fruits of aqueous extract of Sarcocephalus latifolius b. on gentamicin-induced nephrotoxicity in rats. J. Pharm. Pharmacol. Res..

[62] Tomşa A.M., Răchişan A.L., Pandrea S.L., Benea A., Uifălean A., Toma C., Popa R., Pârvu A.E., Junie L.M. (2023). Curcumin and Vitamin C Attenuate Gentamicin-Induced Nephrotoxicity by Modulating Distinctive Reactive Species. Metabolites.

[63] Plavec T., Nemec-Svete A., Butinar J., Tozon N., Prezelj M., Kandel B., Kessler M. (2008). Antioxidant status in canine cancer patients. Acta Vet..

[64] Christo J.S., Rodrigues A.M., Mouro M.G., Cenedeze M.A., de Jesus Simões M., Schor N., Higa E.M.S. (2011). Nitric oxide (NO) is associated with gentamicin (GENTA) nephrotoxicity and the renal function recovery after suspension of GENTA treatment in rats. Nitric Oxide.

[65] Pérez-Torres I., Manzano-Pech L., Rubio-Ruíz M.E., Soto M.E., Guarner-Lans V. (2020). Nitrosative Stress and Its Association with Cardiometabolic Disorders. Molecules.

[66] Szabó C., Ischiropoulos H., Radi R. (2007). Peroxynitrite: Biochemistry, pathophysiology and development of therapeutics. Nat. Rev. Drug Discov..

[67] Metin T.O. (2023). Hepatoprotective effect of pycnogenol in gentamicin-induced liver injury in rats. Ann. Med. Res..

